# Preliminary Investigation of Species Diversity of Rice Hopper Parasitoids in Southeast Asia

**DOI:** 10.3390/insects9010019

**Published:** 2018-02-09

**Authors:** Christina Sann, Franziska Wemheuer, Alexis Beaurepaire, Rolf Daniel, Silvio Erler, Stefan Vidal

**Affiliations:** 1 Agricultural Entomology, Department of Crop Sciences, University of Göttingen, Grisebachstr. 6, 37077 Göttingen, Germany; fwemheu@gwdg.de; 2 Applied Marine and Estuarine Ecology, School of Biological, Earth and Environmental Sciences, The University of New South Wales (UNSW), Sydney, NSW 2052, Australia; 3 Molecular Ecology, Institute of Biology, Martin-Luther-University Halle-Wittenberg, Hoher Weg 4, 06099 Halle (Saale), Germany; alexis.beaurepaire@inra.fr (A.B.); silvio.erler@zoologie.uni-halle.de (S.E.); 4 Abeilles et Environment, INRA PACA, 84914 Avignon CEDEX 9, France; 5 Department of Genomic and Applied Microbiology, Institute of Microbiology and Genetics, University of Göttingen, Grisebachstr. 8, 37077 Göttingen, Germany; rdaniel@gwdg.de

**Keywords:** DNA barcoding, genetic diversity, hymenopteran parasitoids, *Nephotettix*, *Nilaparvata lugens*, rice

## Abstract

Ongoing intensification of rice production systems in Southeast Asia is causing devastating yield losses each year due to rice hoppers. Their continuing development of immunity to resistant rice varieties and pesticide applications further complicates this problem. Hence, there is a high demand for biological control agents of rice hoppers. Egg parasitoid wasps are among the most important natural enemies of rice hoppers, such as *Nilaparvata lugens* and *Nephotettix* spp. However, our knowledge of their diversity is still very limited, due to their small size and the lack of available morphological information. Classifying these parasitoids is the first step to properly understanding their role in the rice agroecosystem. We used traditional morphological identification, as well as DNA sequencing of the 28S rRNA and the COI genes, to investigate the diversity of four important hopper egg parasitoid genera in the Philippines. Parasitoids of the genera *Anagrus*, *Oligosita*, *Gonatocerus*, and *Paracentrobia* were collected in eight study landscapes located in Luzon. Our findings illustrate that characterization of species diversity using morphological and molecular analyses were concordant only for the genus *Paracentrobia*. The genera *Anagrus* and *Gonatocerus* exhibited more genetic diversity than estimated with the morphological analysis, while the opposite was observed for *Oligosita*. This is the first study investigating the molecular diversity of rice hopper parasitoids in the Philippines. More research combining morphological, behavioral, and molecular methods, as well as the establishment of a comprehensive DNA database, are urgently needed to assess the performance and suitability of these organisms as biocontrol agents.

## 1. Introduction

Rice is the main food resource for more than half of the world’s population [1,2]. This makes the rice production system of Asia one of the most important food production systems on Earth [3]. The brown planthopper (BPH; *Nilaparvata lugens*, Stål 1854) and the green leafhopper (GLH; *Nephottetix* spp.) are among the economically most important rice pests. These insects cause immense damage in the Asian rice paddies through xylem sap feeding, resulting in wilting, and subsequently, the death of the rice crops [4], as well as through the transfer of devastating viruses among fields [5,6]. So far, the introduction of rice varieties resistant to BPH and GLH has not been successful, as these pests rapidly adapt to the new varieties [7,8]. The misuse of pesticide applications further enhances the problem by disturbing the rice agroecosystem, which can increase the outbreak risk of hoppers [7,9,10].

As an alternative, integrated pest management (IPM) strategies can be used to maximize the pest’s mortality by enhancing the role of their biological control agents. BPH and GLH are typically attacked by a wide range of natural enemies, such as spiders, predatory bugs, dragonflies, and egg parasitoid wasps from the families Mymaridae and Trichogrammatidae [11]. Among these enemies, parasitoid species are of particular interest. These organisms are very mobile, and can disperse over large distances [12]. The adults feed on pollen, nectar or the honeydew of their sap-feeding hosts, whereas their larvae develop in the hopper eggs and disrupt the hoppers’ life cycles at the earliest possible stage [11]. In two previous studies on egg parasitoids of rice hoppers, egg parasitism levels of more than 60% were observed [13,14]. These findings are supported by Drechsler and Settele [15], who showed that parasitoids can play a major role in controlling hopper pests in rice agroecosystems. 

Despite their importance, knowledge about species composition and diversity of parasitoid wasps in the tropics is still limited (see Nishida, Wongsiri and Wongsiri [16], as well as Gurr et al. [11]). Morphological species identification requires extensive taxonomic expertise, and is hampered by the small size (<1.5 mm) of the wasps as well as the limited amount of literature. To date, molecular information on rice hopper parasitoids from the Philippines is completely lacking. Molecular methods have become a promising tool to resolve species identities [17,18,19]. Different markers, such as the mitochondrial cytochrome c oxidase I (COI) and fragments of the small or large subunits of the ribosomal RNA genes (i.e., 18S or 28S rRNA genes), have been applied to construct hymenopteran and dipteran parasitoid phylogenies [20,21]. However, molecular analyses not only improve species identification, but may also reveal cryptic species [22,23]. Smith et al. [24] suggested combining barcoding with morphology and natural history. A similar conclusion was drawn by Padial and colleagues [25] in their review on an integrative taxonomy for the improvement of species discovery and description.

In a previous study, we investigated parasitoid wasps of *Nilaparvata lugens* (BPH) and *Nephotettix* spp. (GLH) in eight rice production landscapes located in Luzon, Philippines [26]. We found that BPH was parasitized by the Chalcid genera *Anagrus* and *Oligosita,* while GLH was parasitized by the chalcidoid genera *Gonatocerus* and *Paracentrobia*. In the present study, we analyzed the diversity of these genera with traditional morphological and molecular techniques. The present study provides the first molecular identification of parasitoid wasps in rice paddies in the Philippines, combined with a morphological identification based on dichotomous keys.

## 2. Materials and Methods

### 2.1. Study System and Sampling

This study was embedded in the project LEGATO, which focused on sustainable rice production [27]. Parasitoids were collected in eight study landscapes located in the Laguna province, Luzon, Philippines (Supplementary Figure S1). Sampling took place during the rice growing and fallow periods of the dry season from February to June 2013. In brief, rice plants of the variety Taichung Native (1) (TN1) were grown in a greenhouse for 6 weeks, trimmed to three tillers, and covered with small tubular insect cages (85 cm high, 15 cm diameter). Greenhouse cultures of BPH and GLH were reared on TN1, as previously described by Heinrichs et al. [28]. The BPH culture exclusively consisted of *Nilaparvata lugens*, while the GLH culture consisted of *Nephotettix virescens* (Distant) and *Nephotettix nigropictus* (Ståhl). Both hopper populations originated from wild individuals caught in the rice fields of the Laguna Province. Four gravid females of either BPH or GLH were released into each cage for 48 h to lay eggs. Subsequently, the plants were transferred to three different plots per study site to cover the naturally occurring landscape diversity in the Laguna province. Three plants infested with BPH eggs and three plants infested with GLH eggs were distributed randomly within each plot. After 72 h, all plants were returned to the greenhouse and placed back inside separate insect cages. The parasitoids emerged 13–17 days after the field exposure of the eggs. Due to positive phototaxis behavior, parasitoids aggregated in small transparent glass vials, which were placed upside down on top of the sleeved cages. Adult parasitoids were collected daily within this period and immediately stored at −80 °C. The dead parasitoids were identified to genus under a stereo microscope using morphological keys [29,30,31]. In total, 19,455 parasitoids were collected and separated into the four genera *Paracentrobia*, *Gonatocerus, Oligosita,* and *Anagrus* [26].

### 2.2. Preliminary Morphological Identification

Fifty parasitoids from each of the four identified genera (*Paracentrobia*, *Gonatocerus*, *Oligosita,* and *Anagrus*) were randomly selected. The 200 parasitoids were permanently slide-mounted using Faure’s medium, and identified to species level using a microscope. A specialist at the International Rice Research Institute (IRRI) helped with the identification using the key published in Heong and Hardy [30].

### 2.3. DNA Extraction and Amplification

Total genomic DNA was extracted from 267 newly selected whole single female parasitoid individuals (Supplementary Table S2) employing the Phire Animal Tissue Direct PCR Kit (Thermo Scientific, Waltham, MA, USA). Parasitoids were suspended in 20 µL TE buffer (100 mM Tris, 10 mM EDTA). To increase DNA extraction efficiency, 1 µL proteinase K (20 mg mL^−1^) was added. Parasitoid samples were carefully homogenized with sterile micro pestles, and incubated at room temperature overnight.

Two independent gene fragments were amplified from the extracted DNA: one located on the COI subunit I (COI I, amplicon length around 670 bp) and the other one on the expansion regions D2–D3 of the 28S ribosomal subunit (28S-D2/28S-D3, amplicon length approximately 610 bp). The COI region was amplified using the primer pair HCO2198/LCO1490 [32]. The PCR reaction mixture (25 µL) contained 2.5 µL of 10-fold Ex Taq Buffer (Takara Biotechnology, co., LTD, Dalian, China), 25 mM MgCl_2_, 2.5 mM of each of the four dNTPs (deoxynucleotide triphosphates), 10 µM of each primer, 1 U TaKaRa Ex Taq polymerase (Takara Biotechnology), and approximately 25 ng of parasitoid DNA. The following thermal cycling scheme was used: initial denaturation at 94 °C for 3 min, 5 cycles of denaturation at 94 °C for 45 s, annealing at 45 °C for 45 s, followed by elongation at 72 °C for 1 min, and 25 cycles of denaturation at 94 °C for 45 s, annealing at 50 °C for 45 s, followed by another elongation at 72 °C for 1 min. The final extension was carried out at 72 °C for 5 min.

The D2–D3 region of the 28S rRNA gene was amplified as described for the PCR above, using the primers D2-3549 [33] and D2-4068 [34]. The following thermal cycling scheme was used: initial denaturation at 94 °C for 3 min, 30 cycles of denaturation at 94 °C for 45 s, annealing at 58 °C for 45 s, followed by elongation at 72 °C for 1 min. The final extension was carried out at 72 °C for 6 min. Negative controls were performed by using the reaction mixture without template. PCR products were controlled for appropriate size and then purified using the peqGOLD Gel Extraction kit as recommended by the manufacturer (Peqlab, Erlangen, Germany; now VWR).

Sequencing was performed at the Göttingen Genomics Laboratory using an ABI 3730xl system and BigDye terminator chemistry version 3.1 (Thermo Fisher Scientific).

### 2.4. Data Analysis 

Quality control and trimming of the forward and reverse DNA sequences were performed with Gap5 v 1.2.14-r [35]. DNA sequence alignment was performed using MEGA 6 [36]. Additional Chalcidoidea (the super family containing all four genera) sequences were used as reference material. They were retrieved by using the National Centre for Biotechnology Information (NCBI) BLAST tool [37] and the BOLD Identification System (http://www.boldsystems.org) (Supplementary Table S3). The best model to calculate parasitoid phylogeny was determined assuming partial deletion, site coverage cut-off of 95%, and the branch swap filter set to “very strong”. Maximum likelihood trees were generated under the assumption of the best fitting model with 1000 bootstraps. Finally, median-joining networks were constructed using the software NETWORK v. 4.6.1.2 [38]. 

The genetic diversity between and within species was estimated using the reference sequences from the superfamily Chalcidoidea (Supplementary Table S3). The appropriate models for the calculation of the pairwise molecular distances between individuals were determined with MEGA 6. The 28S rRNA distances were calculated based on the K2P + G model. COI distances were calculated based on the T92 + G model. The values were compared using a Kruskal–Wallis test, followed by a Dunn’s post hoc test [39] in R version 3.2.3 [40].

To estimate, whether the genetic diversity of the parasitoids was covered by the sample size used in the analyses, we performed a rarefaction analysis (Supplementary Figures S4 and S5) in R version 3.2.3 using the packages *vegan* [41] and *drc* [42].

### 2.5. Sequence Data Deposition

All sequence data was deposited in the NCBI GenBank under accession number MG785407–MG785510, MG904875–MG904939, MG911990–MG912010.

## 3. Results

### 3.1. Morphological Analysis

In total, 200 parasitoids were morphologically determined. Only one species was found for the genera *Gonatocerus* and *Paracentrobia*, while three species were found for the genera *Anagrus* and *Oligosita*: *Anagrus flaveolus* (11 specimens), *Anagrus optabilis* (6 specimens), *Anagrus frequens* (33 specimens), *Oligosita aesopi* (41 specimens), *Oligosita naias* (7 specimens), *Oligosita shibuyae* (2 specimens), *Gonatocerus orientalis* (50 specimens), and *Paracentrobia andoi* (50 specimens) (Table 1, Supplementary Table S1).

### 3.2. Molecular Analysis

Of the 267 specimens used for the molecular analysis, we successfully sequenced a total of 105 parasitoid samples (Supplementary Table S2). We failed to sequence 162 specimens, due to the poor quality of the extracted DNA, or a failure to extract DNA from the samples. A total of 86 (COI) and 105 (28S) sequences were used to create the maximum likelihood trees. The final dataset for comparison of the two gene fragments included the sequences from 74 parasitoid individuals.

Rarefaction analysis for the local hopper egg parasitoids genetic diversity revealed that the majority of the 28S rRNA gene haplotypes was recovered by the surveying effort (Supplementary Figure S2). By contrast, the rarefaction curve for the COI gene haplotypes was not saturated (Supplementary Figure S3). 

### 3.3. Comparison between the Morphological and Molecular Approach

**Figure 1:**
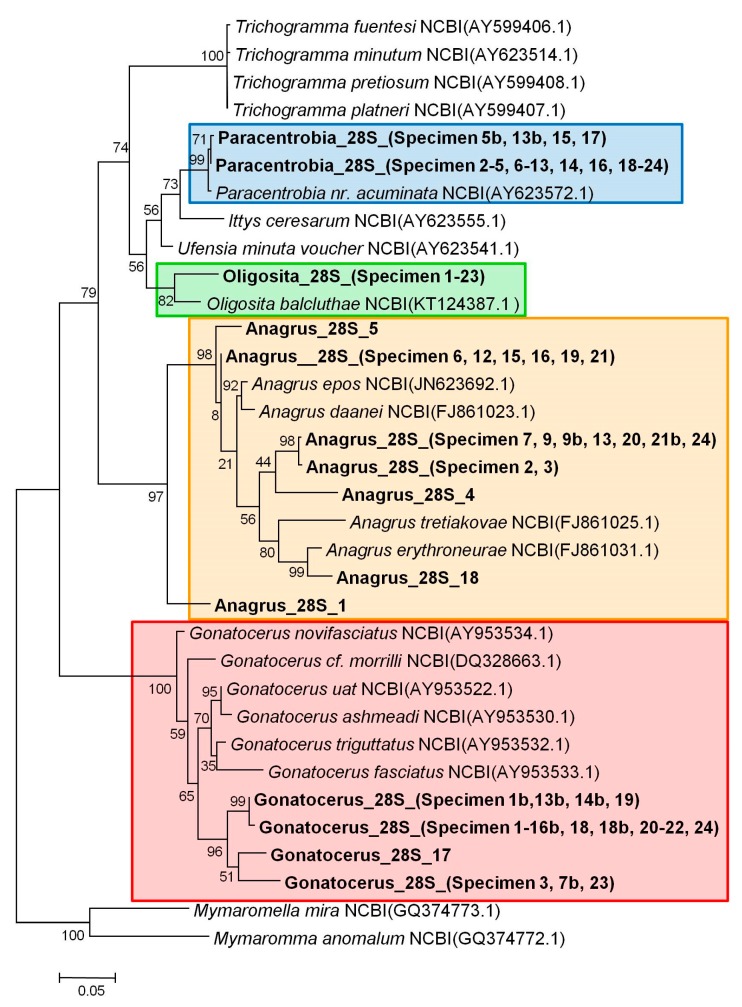
Maximum likelihood tree for the 28S rRNA gene sequences (bootstraps = 1000, TN93 model). Specimens with the same haplotype were pooled. A full list of the specimens used can be found in Supplementary Table S2. Maximum likelihood bootstrap values are given for each node.

**Figure 2:**
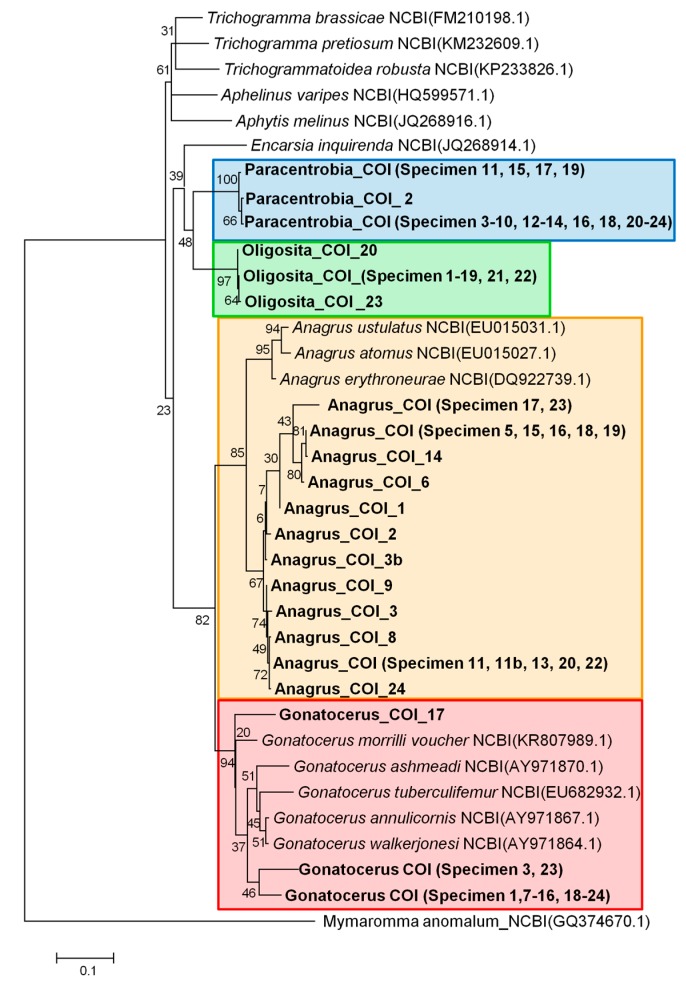
Maximum likelihood tree for the COI sequences (bootstraps = 1000, GTR model). Specimens with the same haplotype were pooled. A full list of the specimens used can be found in Supplementary Table S2. Maximum likelihood bootstrap values are given for each node.

**Figure 3:**
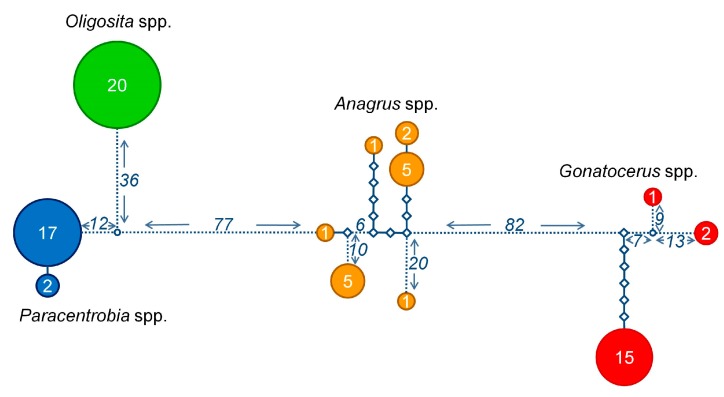
Overall 28S rRNA haplotype distances. Network representing the number of substitutions between the different 28S rRNA gene sequences obtained from different parasitoid genera: *Paracentrobia* (blue); *Oligosita* (green); *Anagrus* (orange); and *Gonatocerus* (red). The sample size for each haplotype is written in the respective circle. Each diamond represents a substitution. The numbers next to the dotted lines are the number of substitutions not represented in detail in the figure.

**Figure 4:**
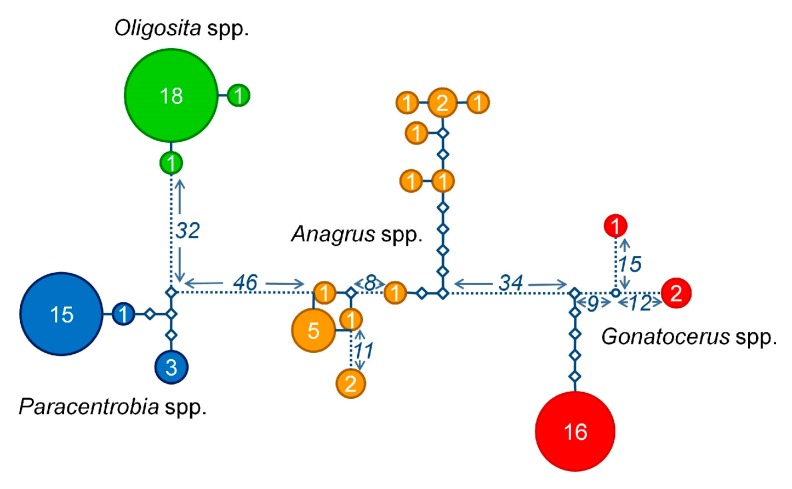
Overall cytochrome c oxidase I (COI) haplotype distance. Network representing the number of substitutions between the different COI sequences obtained from different parasitoid genera: *Paracentrobia* (blue); *Oligosita* (green); *Anagrus* (orange); and *Gonatocerus* (red). The sample size for each haplotype is written in the respective circle. Each diamond represents a substitution. The numbers next to the dotted lines are the number of substitutions not represented in detail in the figure.

The molecular analyses based on COI and 28S rRNA genes revealed that the sequences clearly segregated according to the morphologically pre-assigned genera (Figure 1, Figure 2, Figure 3 and Figure 4, Supplementary Figures S4 and S5). *Oligosita* exhibited the lowest genetic diversity, with three highly similar sequences in the COI gene analysis and only one haplotype in the 28S rRNA gene analysis. In contrast to that, three *Oligosita* species were identified using the morphological approach.

*Paracentrobia* sequences varied only slightly, with two highly similar haplotypes in the 28S rRNA gene analysis and three highly similar haplotypes in the COI gene analysis (Figure 1, Figure 2, Figure 3 and Figure 4, Supplementary Figures S4 and S5). This result indicates that all samples of *Paracentrobia* that we analyzed morphologically and molecularly belonged to a single species.

The sequences for both genes from *Gonatocerus* exhibited more variability compared to the morphological data alone. Three clades were unambiguously identified, with one cluster occurring prevalently (88.2% for the 28S gene, 83.3% for the COI gene) compared to the other two clusters (Figure 1, Figure 2, Figure 3 and Figure 4, Supplementary Figures S4 and S5). *Anagrus* exhibited more variability, and was the most genetically diverse genus: 7 and 12 different haplotypes were identified in the 28S rRNA gene analysis and in the COI gene analysis, respectively (Figure 1 and Figure 2, Supplementary Figures S4 and S5, Supplementary Table S2). 

**Figure 5:**
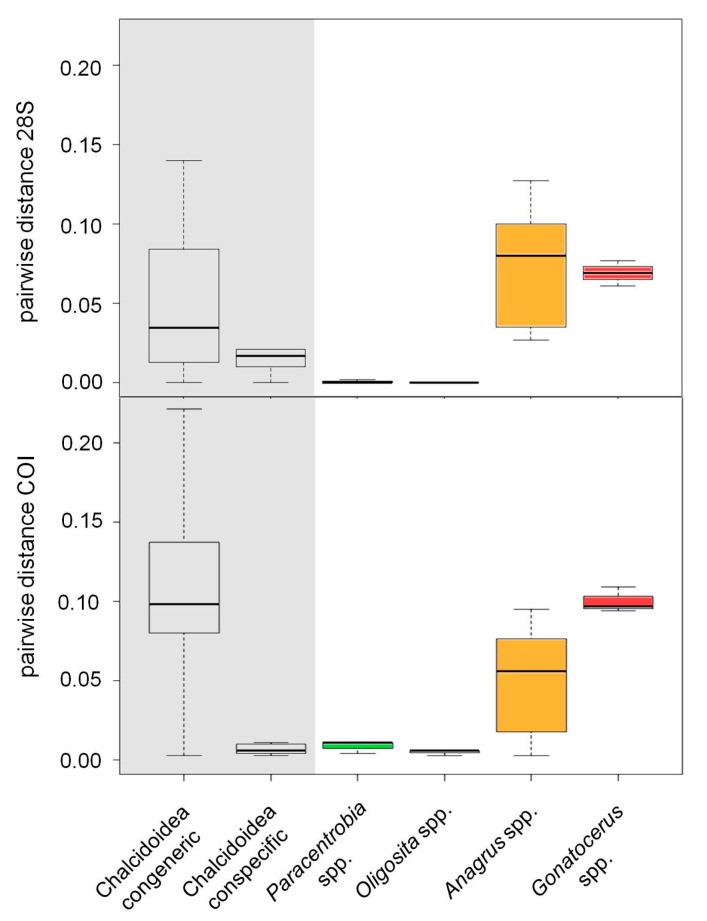
Pairwise genetic distances between individuals from different species of the same genus in the Chalcidoidea (congeneric), different members of the same species in the genus Chalcidoidea (conspecific), and individuals collected in this study. Pairwise distances were calculated by using the K2P+G (Kimura 2-parameter model with gamma distribution) model for the 28S rRNA gene sequences and the T92+G (Tamura 3-parameter model with gamma distribution) model for the COI sequences.

The degrees of genetic diversity found in the genera *Anagrus* and *Gonatocerus* sequences were particularly high. Within group pairwise distances were 0.075 ± 0.012 SE (28S rRNA gene) and 0.050 ± 0.009 SE (COI gene) for *Anagrus*, and 0.069 ± 0.012 SE (28S rRNA gene) and 0.100 ± 0.016 SE (COI gene) for *Gonatocerus*. By contrast, within group pairwise distances were 0.000 ± 0.000 (28S rRNA gene) and 0.005 ± 0.003 SE (COI gene) for *Oligosita*, and 0.001 ± 0.001 SE (28S rRNA gene) and 0.009 ± 0.004 SE (COI gene) for *Paracentrobia* (Figure 5). *Paracentrobia* and *Oligosita* were significantly different from the congeneric Chalcidoidea, but not from the conspecific Chalcidoidea (Figure 5, Table 2). The genetic differences within *Gonatocerus* and *Anagrus* were in the same order of magnitude as the congeneric Chalcidoidea data. 

## 4. Discussion

The molecular analyses of the diversity within the four Chalcidoidea genera studied showed discrepancies with the morphological analysis. This finding is in line with previous studies on hymenopteran parasitoids [23,43,44]. For example, Mottern and Heraty [45] found that one species of the parasitoid previously described as *Cales noacki* were actually ten different *Cales* species. Although previous studies also showed that DNA barcoding can be misleading, resulting in potentially more species than actually exist [46,47], the COI gene, as the classical barcoding gene, has shown its utility in many studies [43,48,49], and constitutes the basis for the BOLD database (www.barcodinglife.org). The 28S rRNA gene is valuable to distinguish among closely related species of Chalcidoidea [50,51,52]. In addition, the 28S rRNA gene is generally more conserved than the COI gene for this group of insects [44,45,53]. This observation is supported by our results, as we found that sampling saturation was only reached for the 28S rRNA gene but not for the COI gene.

Once a reliable and comprehensive DNA database is established, the identification of hopper parasitoids using DNA sequencing will be a vital step towards assessing the performance and suitability of these organisms as biocontrol agents [54,55]. Our study shows that an accurate identification relies on both molecular and morphological techniques. Similar conclusions were drawn previously [21,23,56].

For instance, to date, *Anagrus* species are described as generalist parasitoids that can switch between alternative hopper species in rice agroecosystems [57,58]. Yet, the strong diversity found in the present study by using molecular tools suggests that the *Anagrus* genus is a complex of cryptic species that may be host specialists. This distinction is of high importance to control the hoppers effectively. In addition, the discovery of unknown cryptic species could expand the list of potential biological control agents [45].

Although we were not able to validate the existence of new species of *Anagrus* and *Gonatocerus* by mating tests and/or further morphometric analyses, our data strongly indicates that the two genera include unknown species. The level of within-genus genetic difference, for both 28S rRNA and COI gene sequences among samples from the *Anagrus* and *Gonatocerus* genera, exceeded the threshold of 0.17–2% within-sequence variation, which is generally accepted to delineate individuals of the same species [17,49,59]. According to the literature [17,49,59], genera from the Chalcidoidea superfamily diverge by 5.8–11.25%. Thus, the level of genetic differences we observed for *Anagrus* and *Gonatocerus* suggests that these two genera were composed of individuals from different species. When comparing the genetic analyses to the morphological results, we found strong evidence that there was at least one species of *Anagrus* and *Gonatocerus* that was not detected by the morphological analysis. Interestingly, we observed the opposite for *Oligosita* samples. In the latter genus, it is possible that one or more species were not detected due to the low success rate of DNA amplification. It is also possible that we observed a discrepancy between morphological and molecular analyses, because they were performed on a different set of specimens. As one *Oligosita* species was much more prevalent than the other two in the morphological dataset, we cannot exclude that we analyzed the most common species only, in the molecular analysis.

## 5. Conclusions

In conclusion, our results clearly demonstrate that molecular identification should be used in combination with morphological methods and mating tests for assessing the diversity of rice hopper parasitoids. However, further studies using an integrative approach are needed to cover the whole diversity of parasitoids, as well as to find sustainable solutions to problems caused by BPH and GLH. To validate the potential application of parasitoid wasps as biocontrol agents, it is of crucial importance to have a comprehensive knowledge on their ecology and diversity. We hope that this study will encourage further research by providing the first barcodes for egg parasitoid species from rice paddies in Southeast Asia.

## Figures and Tables

**Table 1:** Species identified by the morphological analysis using 50 specimens per genera (GLH: green leafhopper, BPH: brown planthopper).

Species	Host	Examined Individuals
*Paracentrobia andoi*	GLH	50
*Oligosita aesopi*	BPH	41
*Oligosita naias*	BPH	7
*Oligosita shibuyae*	BPH	2
*Anagrus frequens*	BPH	33
*Anagrus optabilis*	BPH	6
*Anagrus flaveolus*	BPH	11
*Gonatocerus orientalis*	GLH	50

**Table 2:** Results of the Dunn’s test (*p*) with degrees of freedom (*df*) for pairwise comparison of the pairwise genetic distances calculated within the parasitoid genera examined in this study, and the pairwise genetic differences calculated for the congeneric and conspecific Chalcidoidea sequences (n.s. stands for not significant).

**Gene**	**Parasitoid Group**	***Paracentrobia* spp.**		***Oligosita* spp.**		***Anagrus* spp.**		***Gonatocerus* spp.**	
		*p*	*df*	*p*	*df*	*p*	*df*	*p*	*df*
28S gene	Chalcidoidea congeneric	0.003	2	0.003	2	0.021	2	n.s.	2
	Chalcidoidea conspecific	n.s.	2	n.s.	2	<0.001	2	0.05	2
COI gene	Chalcidoidea congeneric	0.017	2	0.002	2	<0.001	2	n.s.	2
	Chalcidoidea conspecific	n.s.	2	n.s.	2	<0.001	2	0.006	2

## Supplementary Materials

The following are available online at http://www.mdpi.com/2075-4450/9/1/19/s1, Figure S1: Distribution of the eight study sites throughout the Laguna province, Figure S2: Rarefaction curve for the 28S rRNA gene, Figure S3: Rarefaction curve for the COI gene, Figure S4: Maximum likelihood tree for the 28S rRNA sequences, Figure S5: Maximum likelihood tree for the COI sequences, Table S1: Morphometric results for parasitoids throughout the Laguna province, Philippines, Table S2: Sequences obtained from parasitoids of the rice fields in the Philippines, Table S3: Sequences retrieved from the NCBI GenBank.

## References

[1] FAO. http://www.fao.org/faostat/en/#home.

[2] IRRI—Trends in Global Rice Consumption. http://irri.org/rice-today/trends-in-global-rice-consumption.

[3] Gurr G.M., Lu Z., Zheng X., Xu H., Zhu P., Chen G., Yao X., Cheng J., Zhu Z., Catindig J.L. (2016). Multi-country evidence that crop diversification promotes ecological intensification of agriculture. Nat. Plants.

[4] Heong K.L., Aquino G.-B., Barrion A.T. (1992). Population dynamics of plant- and leafhoppers and their natural enemies in rice ecosystems in the Philippines. Crop Prot..

[5] Ling K.C. (1972). Rice Virus Diseases.

[6] Cabauatan P.Q., Cabunagan R.C., Choi I.-R., Heong K.L., Hardy B. (2009). Rice viruses transmitted by the brown planthopper *Nilaparvata lugens* Stal. Planthoppers: New Threats to the Sustainability of Intensive Rice Production Systems in Asia.

[7] Catindig J.L., Arida G.S., Baehaki S.E., Bentur J.S., Cuong L.Q., Norowi M., Rattanakarn W., Sriratanasak W., Xia J., Lu Z., Heong K.L., Hardy B. (2009). Situations of planthoppers in Asia. Planthoppers: New Threats to the Sustainability of Intensive Rice Production Systems in Asia.

[8] Seo B.Y., Jung J.K., Choi B.-R., Park H.M., Lee B.H. (2009). Resistance-breaking ability and feeding behavior of the brown planthopper *Nilaparvata lugens*, recently collected in Korea. Planthoppers: New Threats to the Sustainability of Intensive Rice Production Systems in Asia.

[9] Heong K.L., Schoenly K.G. (1998). Impact of insecticides on herbivore-natural enemy communities in tropical rice ecosystems. Ecotoxicology.

[10] Heong K.L., Heong K.L., Hardy B. (2009). Are planthopper problems caused by a breakdown in ecosystem services?. Planthoppers: New Threats to the Sustainability of Intensive Rice Production Systems in Asia.

[11] Gurr G.M., Liu J., Read D.M.Y., Catindig J.L.A., Cheng J.A., Lan L.P., Heong K.L. (2011). Parasitoids of Asian rice planthopper (Hemiptera: Delphacidae) pests and prospects for enhancing biological control by ecological engineering. Ann. Appl. Biol..

[12] Antolin M.F., Strong D.R. (1987). Long-distance dispersal by a parasitoid (*Anagrus delicatus*, Mymaridae) and its host. Oecologia.

[13] Fowler S.V., Claridge M.F., Morgan J.C., Peries I.D.R., Nugaliyadde L. (1991). Egg mortality of the brown planthopper, *Nilaparvata lugens* (Homoptera: Delphacidae) and green leafhoppers, *Nephotettix* spp. (Homoptera: Cicadellidae), on rice in Sri Lanka. Bull. Entomol. Res..

[14] Watanabe T., Wada T., Salleh N.M.N. (1992). Parasitic Activities of Egg Parasitoids on the rice planthoppers, *Nilaparvata lugens* (STAL) and *Sogatella furcifera* (HORVATH) (Homoptera: Delphacidae), in the Muda Area, Peninsular Malaysia. Appl. Entomol. Zool..

[15] Drechsler M., Settele J. (2001). Predator–prey interactions in rice ecosystems: Effects of guild composition, trophic relationships, and land use changes—A model study exemplified for Philippine rice terraces. Ecol. Model..

[16] Nishida T., Wongsiri T., Wongsiri N. (1976). Species composition, population trends and egg parasitism of planthopper and leafhopper rice pests of Thailand. Plant Prot. Bull. FAO.

[17] Chesters D., Wang Y., Yu F., Bai M., Zhang T.-X., Hu H.-Y., Zhu C.-D., Li C.-D., Zhang Y.-Z. (2012). The integrative taxonomic approach reveals host specific species in an encyrtid parasitoid species Complex. PLoS ONE.

[18] Hebert P.D.N., Penton E.H., Burns J.M., Janzen D.H., Hallwachs W. (2004). Ten species in one: DNA barcoding reveals cryptic species in the Neotropical skipper butterfly *Astraptes fulgerator*. Proc. Natl. Acad. Sci. USA.

[19] Smith M.A., Rodriguez J.J., Whitfield J.B., Deans A.R., Janzen D.H., Hallwachs W., Hebert P.D.N. (2008). Extreme diversity of tropical parasitoid wasps exposed by iterative integration of natural history, DNA barcoding, morphology, and collections. Proc. Natl. Acad. Sci. USA.

[20] Schwarzfeld M.D., Broad G.R., Sperling F.A.H. (2016). Molecular phylogeny of the diverse parasitoid wasp genus *Ophion Fabricius* (Hymenoptera: Ichneumonidae: Ophioninae): Phylogeny of *Ophion*. Syst. Entomol..

[21] Munro J.B., Heraty J.M., Burks R.A., Hawks D., Mottern J., Cruaud A., Rasplus J.-Y., Jansta P. (2011). A molecular phylogeny of the Chalcidoidea (Hymenoptera). PLoS ONE.

[22] Smith M.A., Woodley N.E., Janzen D.H., Hallwachs W., Hebert P.D.N. (2006). DNA barcodes reveal cryptic host-specificity within the presumed polyphagous members of a genus of parasitoid flies (Diptera: Tachinidae). Proc. Natl. Acad. Sci. USA.

[23] Zhou Q.-S., Xi Y.-Q., Yu F., Zhang X., Li X.-J., Liu C.-L., Niu Z.-Q., Zhu C.-D., Qiao G.-X., Zhang Y.-Z. (2014). Application of DNA barcoding to the identification of Hymenoptera parasitoids from the soybean aphid (*Aphis glycines*) in China: DNA barcoding to identify parasitoids from Aphis glycines in China. Insect Sci..

[24] Smith M.A., Wood D.M., Janzen D.H., Hallwachs W., Hebert P.D.N. (2007). DNA barcodes affirm that 16 species of apparently generalist tropical parasitoid flies (Diptera, Tachinidae) are not all generalists. Proc. Natl. Acad. Sci. USA.

[25] Padial J.M., Miralles A., De la Riva I., Vences M. (2010). The integrative future of taxonomy. Front. Zool..

[26] Sann C., Theodorou P., Heong K.L., Villareal S., Settele J., Vidal S., Westphal C. (2018). Hopper parasitoids do not significantly benefit from non-crop habitats in rice production landscapes. Agric. Ecosyst. Environ..

[27] Settele J., Spangenberg J.H., Heong K.L., Burkhard B., Bustamante J.V., Cabbigat J., Van Chien H., Escalada M., Grescho V., Hai L.H. (2015). Agricultural landscapes and ecosystem services in South-East Asia—The LEGATO-Project. Basic Appl. Ecol..

[28] Heinrichs E.A., Medrano F.G., Rapusas H.R. (1985). Genetic Evaluation for Insect in Rice.

[29] Shepard B.M., Barrion A.T., Listinger J.A. (1987). Friends of the Rice Farmer. Helpful Insects, Spiders and Pathogens.

[30] Dupo A.L., Barrion A.T., Heong K.L., Hardy B. (2009). Taxonomy and general biology of delphacid planthoppers in rice agroecosystems. Planthoppers: New Threats to the Sustainability of Intensive Rice Production Systems in Asia.

[31] Heinrichs E.A. (1994). Biology and Management of Rice Insects.

[32] Folmer O., Black M., Hoeh W., Lutz R., Vrijenhoek R. (1994). DNA primers for amplification of mitochondrial cytochrome c oxidase subunit I from diverse metazoan invertebrates. Mol. Mar. Biol. Biotechnol..

[33] Campbell B.C., Steffen-Campbell J.D., Werren J.H. (1993). Phylogeny of the Nasonia species complex (Hymenoptera: Pteromalidae) inferred from an internal transcribed spacer (ITS2) and 28S rDNA sequences. Insect Mol. Biol..

[34] Campbell B.C., Heraty J.M., Rasplus J.Y., Chan K., Steffen-Campbell J.D., Babcock C., Heong K.L., Hardy B. (2000). Molecular systematic of the Chalcidoidea using 28S-D2 rDNA. Hymenoptera: Evolution, Biodiversity and Biological Control.

[35] Bonfield J.K., Whitwham A. (2010). Gap5—Editing the billion fragment sequence assembly. Bioinformatics.

[36] Tamura K., Dudley J., Nei M., Kumar S. (2007). MEGA4: Molecular Evolutionary Genetics Analysis (MEGA) software version 4.0. Mol. Biol. Evol..

[37] Altschul S.F., Gish W., Miller W., Myers E.W., Lipman D.J. (1990). Basic local alignment search tool. J. Mol. Biol..

[38] Bandelt H.J., Forster P., Rohl A. (1999). Median-joining networks for inferring intraspecific phylogenies. Mol. Biol. Evol..

[39] Dunn O.J. (1964). Multiple comparisons using rank sums. Technometrics.

[40] R Development Core Team (2013). The R Foundation for Statistical Computing, Ver. 3.0.2.

[41] Dixon P. (2003). VEGAN, a package of R functions for community ecology. J. Veg. Sci..

[42] Ritz C., Baty F., Streibig J.C., Gerhard D. (2015). Dose-response analysis using R. PLoS ONE.

[43] Smith M., Fernández-Triana J.L., Eveleigh E., Gómez J., Guclu C., Hallwachs W., Hebert P.D.N., Hrcek J., Huber J.T., Janzen D. (2013). DNA barcoding and the taxonomy of Microgastrinae wasps (Hymenoptera, Braconidae): Impacts after 8 years and nearly 20000 sequences. Mol. Ecol. Resour..

[44] Kenyon S.G., Buerki S., Hansson C., Alvarez N., Benrey B. (2015). Uncovering cryptic parasitoid diversity in Horismenus (Chalcidoidea, Eulophidae). PLoS ONE.

[45] Mottern J.L., Heraty J.M. (2014). Revision of the *Cales noacki* species complex (Hymenoptera, Chalcidoidea, Aphelinidae). Syst. Entomol..

[46] Brower A.V.Z. (2006). Problems with DNA barcodes for species delimitation: “Ten species” of *Astraptes fulgerator* reassessed (Lepidoptera: Hesperiidae). Syst. Biodivers..

[47] Collins R.A., Cruickshank R.H. (2013). The seven deadly sins of DNA barcoding. Mol. Ecol. Resour..

[48] Gutiérrez-Arellano D., Gutiérrez-Arellano C., Zaldívar-Riverón A. (2015). DNA Barcoding of the parasitoid wasp subfamily Doryctinae (Hymenoptera: Braconidae) from Chamela, Mexico. Biodivers. Data J..

[49] Hebert P.D.N., Cywinska A., Ball S.L., deWaard J.R. (2003). Biological identifications through DNA barcodes. Proc. R. Soc. B Biol. Sci..

[50] Babcock C.S., Heraty J.M., De Barro P.J., Driver F., Schmidt S. (2001). Preliminary phylogeny of *Encarsia Förster* (Hymenoptera: Aphelinidae) based on morphology and 28S rDNA. Mol. Phylogenet. Evol..

[51] Manzari S., Polaszek A., Belshaw R., Quicke D.L.J. (2002). Morphometric and molecular analysis of the *Encarsia inaron* species-group (Hymenoptera: Aphelinidae), parasitoids of whiteflies (Hemiptera: Aleyrodidae). Bull. Entomol. Res..

[52] Triapitsyn S.V., Vickerman D.B., Heraty J.M., Logarzo G.A. (2006). A new species of *Gonatocerus* (Hymenoptera: Mymaridae) parasitic On *Proconiine Sharpshooters* (Hemiptera: Cicadellidae) in the New World. Zootaxa.

[53] Gillespie J.J. (2005). A secondary structural model of the 28S rRNA expansion segments D2 and D3 for chalcidoid wasps (Hymenoptera: Chalcidoidea). Mol. Biol. Evol..

[54] Greenstone M.H. (2006). Molecular methods for assessing insect parasitism. Bull. Entomol. Res..

[55] Gariepy T.D., Kuhlmann U., Gillott C., Erlandson M. (2007). Parasitoids, predators and PCR: The use of diagnostic molecular markers in biological control of Arthropods. J. Appl. Entomol..

[56] Desneux N., Starý P., Delebecque C.J., Gariepy T.D., Barta R.J., Hoelmer K.A., Heimpel G.E. (2009). Cryptic species of parasitoids attacking the soybean aphid (Hemiptera: Aphididae) in Asia: Binodoxys communis and binodoxys koreanus (Hymenoptera: Braconidae: Aphidiinae). Ann. Entomol. Soc. Am..

[57] Zheng X., Yu X., Lu Z., Chen J., Xu H., Ju R. (2003). Parasitization adaptability of *Anagrus optabilis* on *Nilaparvata lugens*. Chin. J. Biol. Control.

[58] Zheng X., Lu Y., Zhu P., Zhang F., Tian J., Xu H., Chen G., Nansen C., Lu Z. (2017). Use of banker plant system for sustainable management of the most important insect pest in rice fields in China. Sci. Rep..

[59] Meier R., Zhang G., Ali F. (2008). The use of mean instead of smallest interspecific distances exaggerates the size of the “Barcoding Gap” and leads to misidentification. Syst. Biol..

