# Supplementary material for: Preliminary Investigation of Species Diversity of Rice Hopper Parasitoids in Southeast Asia
Source: Insects. 2018 Feb 9;9(1):19. doi: 10.3390/insects9010019 (PMC5872284; doi:10.3390/insects9010019)
Supplement: Supplementary file 1 [file insects-09-00019-s001.docx]

**Supporting Information: Preliminary Investigation of Species Diversity of Rice Hopper Parasitoids in Southeast Asia**

**Table S1.** Species found, according to morphometric analysis, throughout the Laguna province, Philippines. (GLH: *Paracentrobia andoi*, *Gonatocerus orientalis*; BPH: *Oligosita aesopi, Oligosita naias, Oligosita shibuyae, Anagrus frequens, Anagrus optabilis, Anagrus flaveolus).*

| **Species** | **Host** | **Examined individuals** | **Site** | **Co-Ordinates** |
| --- | --- | --- | --- | --- |
| *Paracentrobia andoi* | GLH | 3 | S1B | N 14.22857 E121.33542 |
| *Paracentrobia andoi* | GLH | 1 | S2A | N 14.11583 E 121.41298 |
| *Paracentrobia andoi* | GLH | 4 | S2C | N 14.11540 E 121.41070 |
| *Paracentrobia andoi* | GLH | 1 | S3A | N 14.21248 E 121.33754 |
| *Paracentrobia andoi* | GLH | 7 | S3B | N 14.21577 E 121.33724 |
| *Paracentrobia andoi* | GLH | 3 | S3C | N 14.21740 E 121.33510 |
| *Paracentrobia andoi* | GLH | 2 | S4B | N 14.13942 E 121.39849 |
| *Paracentrobia andoi* | GLH | 2 | S4C | N 14.13856 E 121.40070 |
| *Paracentrobia andoi* | GLH | 4 | S5A | N 14.18689 E 121.36572 |
| *Paracentrobia andoi* | GLH | 2 | S5B | N 14.18674 E 121.36550 |
| *Paracentrobia andoi* | GLH | 1 | S6A | N 14.207742 E 121.332669 |
| *Paracentrobia andoi* | GLH | 6 | S6C | N 14.209896 E 121.331937 |
| *Paracentrobia andoi* | GLH | 2 | S7A | N 14.184159 E 121.362021 |
| *Paracentrobia andoi* | GLH | 5 | S7B | N 14.184277 E 121.361807 |
| *Paracentrobia andoi* | GLH | 2 | S8B | N 14.147272 E 121.323637 |
| *Paracentrobia andoi* | GLH | 5 | S8C | N 14.148391 E 121.322570 |
|  |  |  |  |  |
| *Oligosita aesopi* | BPH | 4 | S1B | N 14.22857 E121.33542 |
| *Oligosita aesopi* | BPH | 2 | S2A | N 14.11583 E 121.41298 |
| *Oligosita aesopi* | BPH | 1 | S2C | N 14.11540 E 121.41070 |
| *Oligosita aesopi* | BPH | 1 | S3A | N 14.21248 E 121.33754 |
| *Oligosita aesopi* | BPH | 2 | S3B | N 14.21577 E 121.33724 |
| *Oligosita aesopi* | BPH | 3 | S3C | N 14.21740 E 121.33510 |
| *Oligosita aesopi* | BPH | 2 | S4B | N 14.13942 E 121.39849 |
| *Oligosita aesopi* | BPH | 1 | S4C | N 14.13856 E 121.40070 |
| *Oligosita aesopi* | BPH | 3 | S5A | N 14.18689 E 121.36572 |
| *Oligosita aesopi* | BPH | 1 | S5B | N 14.18674 E 121.36550 |
| *Oligosita aesopi* | BPH | 2 | S6A | N 14.207742 E 121.332669 |
| *Oligosita aesopi* | BPH | 3 | S6C | N 14.209896 E 121.331937 |
| *Oligosita aesopi* | BPH | 1 | S7A | N 14.184159 E 121.362021 |
| *Oligosita aesopi* | BPH | 8 | S7B | N 14.184277 E 121.361807 |
| *Oligosita aesopi* | BPH | 5 | S8B | N 14.147272 E 121.323637 |
| *Oligosita aesopi* | BPH | 2 | S8C | N 14.148391 E 121.322570 |
| *Oligosita naias* | BPH | 2 | S3B | N 14.21577 E 121.33724 |
| *Oligosita naias* | BPH | 3 | S6C | N 14.209896 E 121.331937 |
| *Oligosita naias* | BPH | 2 | S8C | N 14.148391 E 121.322570 |
| *Oligosita shibuyae* | BPH | 2 | S1B | N 14.22857 E121.33542 |
|  |  |  |  |  |
| *Anagrus flaveolus* | BPH | 2 | S2C | N 14.11540 E 121.41070 |
| *Anagrus flaveolus* | BPH | 1 | S4B | N 14.13942 E 121.39849 |
| *Anagrus flaveolus* | BPH | 5 | S5B | N 14.18674 E 121.36550 |
| *Anagrus flaveolus* | BPH | 1 | S6C | N 14.209896 E 121.331937 |
| *Anagrus flaveolus* | BPH | 2 | S7A | N 14.184159 E 121.362021 |
| *Anagrus frequens* | BPH | 1 | S1B | N 14.22857 E121.33542 |
| *Anagrus frequens* | BPH | 5 | S2A | N 14.11583 E 121.41298 |
| *Anagrus frequens* | BPH | 3 | S3B | N 14.21577 E 121.33724 |
| *Anagrus frequens* | BPH | 1 | S3C | N 14.21740 E 121.33510 |
| *Anagrus frequens* | BPH | 2 | S4B | N 14.13942 E 121.39849 |
| *Anagrus frequens* | BPH | 2 | S4C | N 14.13856 E 121.40070 |
| *Anagrus frequens* | BPH | 7 | S5A | N 14.18689 E 121.36572 |
| *Anagrus frequens* | BPH | 3 | S6A | N 14.207742 E 121.332669 |
| *Anagrus frequens* | BPH | 1 | S6C | N 14.209896 E 121.331937 |
| *Anagrus frequens* | BPH | 2 | S7A | N 14.184159 E 121.362021 |
| *Anagrus frequens* | BPH | 5 | S7B | N 14.184277 E 121.361807 |
| *Anagrus frequens* | BPH | 1 | S8B | N 14.147272 E 121.323637 |
| *Anagrus optabilis* | BPH | 2 | S2A | N 14.11583 E 121.41298 |
| *Anagrus optabilis* | BPH | 1 | S6C | N 14.209896 E 121.331937 |
| *Anagrus optabilis* | BPH | 3 | S7A | N 14.184159 E 121.362021 |
|  |  |  |  |  |
| *Gonatocerus orientalis* | GLH | 1 | S1B | N 14.22857 E121.33542 |
| *Gonatocerus orientalis* | GLH | 5 | S2A | N 14.11583 E 121.41298 |
| *Gonatocerus orientalis* | GLH | 3 | S2C | N 14.11540 E 121.41070 |
| *Gonatocerus orientalis* | GLH | 2 | S3A | N 14.21248 E 121.33754 |
| *Gonatocerus orientalis* | GLH | 1 | S3B | N 14.21577 E 121.33724 |
| *Gonatocerus orientalis* | GLH | 4 | S3C | N 14.21740 E 121.33510 |
| *Gonatocerus orientalis* | GLH | 2 | S4B | N 14.13942 E 121.39849 |
| *Gonatocerus orientalis* | GLH | 3 | S4C | N 14.13856 E 121.40070 |
| *Gonatocerus orientalis* | GLH | 5 | S5A | N 14.18689 E 121.36572 |
| *Gonatocerus orientalis* | GLH | 2 | S5B | N 14.18674 E 121.36550 |
| *Gonatocerus orientalis* | GLH | 9 | S6A | N 14.207742 E 121.332669 |
| *Gonatocerus orientalis* | GLH | 1 | S6C | N 14.209896 E 121.331937 |
| *Gonatocerus orientalis* | GLH | 1 | S7A | N 14.184159 E 121.362021 |
| *Gonatocerus orientalis* | GLH | 2 | S7B | N 14.184277 E 121.361807 |
| *Gonatocerus orientalis* | GLH | 8 | S8B | N 14.147272 E 121.323637 |
| *Gonatocerus orientalis* | GLH | 1 | S8C | N 14.148391 E 121.322570 |

**Table S2**. Sequences obtained from parasitoids of the rice fields in the Philippines.

| **Sequence ID** | **Network** | **Insect host** | **Genus** | **Gen** | **Haplotype** | **Genbank** | **Location abbr.** | **Co-Ordinates** |
| --- | --- | --- | --- | --- | --- | --- | --- | --- |
| **Paracentrobia_28S_2** | **yes** | GLH | Paracentrobia | 28S | P28S_1 | MG785460 | S3C | N 14.21740 E 121.33510 |
| **Paracentrobia_28S_3** | **yes** | GLH | Paracentrobia | 28S | P28S_1 | MG785461 | S2C | N 14.11540 E 121.41070 |
| Paracentrobia_28S_3b | no | GLH | Paracentrobia | 28S | P28S_1 | MG785462 | S3C | N 14.21740 E 121.33510 |
| **Paracentrobia_28S_5** | no | GLH | Paracentrobia | 28S | P28S_1 | MG785463 | S1C | N 14.22862 E 121.33980 |
| Paracentrobia_28S_5b | no | GLH | Paracentrobia | 28S | P28S_2 | MG785464 | S4C | N 14.13856 E 121.40070 |
| **Paracentrobia_28S_6** | **yes** | GLH | Paracentrobia | 28S | P28S_1 | MG785465 | S4C | N 14.13856 E 121.40070 |
| **Paracentrobia_28S_7** | **yes** | GLH | Paracentrobia | 28S | P28S_1 | MG785466 | S1C | N 14.22862 E 121.33980 |
| **Paracentrobia_28S_8** | **yes** | GLH | Paracentrobia | 28S | P28S_1 | MG785467 | S3C | N 14.21740 E 121.33510 |
| Paracentrobia_28S_8b | no | GLH | Paracentrobia | 28S | P28S_1 | MG785468 | S7C | N 14.185338 E 121.360673 |
| **Paracentrobia_28S_9** | **yes** | GLH | Paracentrobia | 28S | P28S_1 | MG785469 | S7B | N 14.184277 E 121.361807 |
| Paracentrobia_28S_9b | no | GLH | Paracentrobia | 28S | P28S_1 | MG785470 | S7B | N 14.184277 E 121.361807 |
| Paracentrobia_28S_10 | no | GLH | Paracentrobia | 28S | P28S_1 | MG785471 | S8B | N 14.147272 E 121.323637 |
| Paracentrobia_28S_10b | no | GLH | Paracentrobia | 28S | P28S_1 | MG785472 | S3B | N 14.21577 E 121.33724 |
| **Paracentrobia_28S_11** | **yes** | GLH | Paracentrobia | 28S | P28S_1 | MG785473 | S1B | N 14.22857 E121.33542 |
| Paracentrobia_28S_11b | no | GLH | Paracentrobia | 28S | P28S_1 | MG785474 | S1B | N 14.22857 E121.33542 |
| **Paracentrobia_28S_12** | **yes** | GLH | Paracentrobia | 28S | P28S_1 | MG785475 | S3B | N 14.21577 E 121.33724 |
| **Paracentrobia_28S_13** | **yes** | GLH | Paracentrobia | 28S | P28S_2 | MG785476 | S1B | N 14.22857 E121.33542 |
| Paracentrobia_28S_13b | no | GLH | Paracentrobia | 28S | P28S_1 | MG785477 | S1B | N 14.22857 E121.33542 |
| **Paracentrobia_28S_14** | **yes** | GLH | Paracentrobia | 28S | P28S_1 | MG785478 | S8B | N 14.147272 E 121.323637 |
| **Paracentrobia_28S_15** | **yes** | GLH | Paracentrobia | 28S | P28S_2 | MG785479 | S1B | N 14.22857 E121.33542 |
| Paracentrobia_28S_15b | no | GLH | Paracentrobia | 28S | P28S_1 | MG785480 | S1B | N 14.22857 E121.33542 |
| **Paracentrobia_28S_16** | **yes** | GLH | Paracentrobia | 28S | P28S_1 | MG785481 | S5B | N 14.18674 E 121.36550 |
| Paracentrobia_28S_16b | no | GLH | Paracentrobia | 28S | P28S_1 | MG785482 | S6B | N 14.207981 E 121.332345 |
| Paracentrobia_28S_16c | no | GLH | Paracentrobia | 28S | P28S_1 | MG785483 | S6B | N 14.207981 E 121.332345 |
| **Paracentrobia_28S_17** | **yes** | GLH | Paracentrobia | 28S | P28S_2 | MG785484 | S1A | N 14.22921 E 121.33537 |
| Paracentrobia_28S_17b | no | GLH | Paracentrobia | 28S | P28S_1 | MG785485 | S1A | N 14.22921 E 121.33537 |
| **Paracentrobia_28S_18** | **yes** | GLH | Paracentrobia | 28S | P28S_1 | MG785486 | S5A | N 14.18689 E 121.36572 |
| **Paracentrobia_28S_21** | **yes** | GLH | Paracentrobia | 28S | P28S_1 | MG785487 | S1A | N 14.22921 E 121.33537 |
| **Paracentrobia_28S_22** | **yes** | GLH | Paracentrobia | 28S | P28S_1 | MG785488 | S1A | N 14.22921 E 121.33537 |
| **Paracentrobia_28S_23** | **yes** | GLH | Paracentrobia | 28S | P28S_1 | MG785489 | S1A | N 14.22921 E 121.33537 |
| **Paracentrobia_28S_24** | **yes** | GLH | Paracentrobia | 28S | P28S_1 | MG785490 | S1A | N 14.22921 E 121.33537 |
|  |  |  |  |  |  |  |  |  |
| **Paracentrobia_COI_2** | **yes** | GLH | Paracentrobia | COI | PCOI_1 | MG904918 | S3C | N 14.21740 E 121.33510 |
| **Paracentrobia_COI_3** | **yes** | GLH | Paracentrobia | COI | PCOI_2 | MG904919 | S2C | N 14.11540 E 121.41070 |
| Paracentrobia_COI_4 | no | GLH | Paracentrobia | COI | PCOI_2 | MG904920 | S8C | N 14.148391 E 121.322570 |
| **Paracentrobia_COI_5** | **yes** | GLH | Paracentrobia | COI | PCOI_2 | MG904921 | S1C | N 14.22862 E 121.33980 |
| **Paracentrobia_COI_6** | **yes** | GLH | Paracentrobia | COI | PCOI_2 | MG904922 | S4C | N 14.13856 E 121.40070 |
| **Paracentrobia_COI_7** | **yes** | GLH | Paracentrobia | COI | PCOI_2 | MG904923 | S1C | N 14.22862 E 121.33980 |
| **Paracentrobia_COI_8** | **yes** | GLH | Paracentrobia | COI | PCOI_2 | MG904924 | S3C | N 14.21740 E 121.33510 |
| **Paracentrobia_COI_9** | **yes** | GLH | Paracentrobia | COI | PCOI_2 | MG904925 | S7B | N 14.184277 E 121.361807 |
| **Paracentrobia_COI_11** | **yes** | GLH | Paracentrobia | COI | PCOI_3 | MG904926 | S1B | N 14.22857 E121.33542 |
| **Paracentrobia_COI_12** | **yes** | GLH | Paracentrobia | COI | PCOI_2 | MG904927 | S3B | N 14.21577 E 121.33724 |
| **Paracentrobia_COI_13** | **yes** | GLH | Paracentrobia | COI | PCOI_2 | MG904928 | S1B | N 14.22857 E121.33542 |
| **Paracentrobia_COI_14** | **yes** | GLH | Paracentrobia | COI | PCOI_2 | MG904929 | S8B | N 14.147272 E 121.323637 |
| **Paracentrobia_COI_15** | **yes** | GLH | Paracentrobia | COI | PCOI_3 | MG904930 | S1B | N 14.22857 E121.33542 |
| **Paracentrobia_COI_16** | **yes** | GLH | Paracentrobia | COI | PCOI_2 | MG904931 | S5B | N 14.18674 E 121.36550 |
| **Paracentrobia_COI_17** | **yes** | GLH | Paracentrobia | COI | PCOI_3 | MG904932 | S1A | N 14.22921 E 121.33537 |
| **Paracentrobia_COI_18** | **yes** | GLH | Paracentrobia | COI | PCOI_2 | MG904933 | S5A | N 14.18689 E 121.36572 |
| Paracentrobia_COI_19 | no | GLH | Paracentrobia | COI | PCOI_3 | MG904934 | S1A | N 14.22921 E 121.33537 |
| Paracentrobia_COI_20 | no | GLH | Paracentrobia | COI | PCOI_2 | MG904935 | S8A | N 14.146850 E 121.323753 |
| **Paracentrobia_COI_21** | **yes** | GLH | Paracentrobia | COI | PCOI_2 | MG904936 | S1A | N 14.22921 E 121.33537 |
| **Paracentrobia_COI_22** | **yes** | GLH | Paracentrobia | COI | PCOI_2 | MG904937 | S1A | N 14.22921 E 121.33537 |
| **Paracentrobia_COI_23** | **yes** | GLH | Paracentrobia | COI | PCOI_2 | MG904938 | S1A | N 14.22921 E 121.33537 |
| **Paracentrobia_COI_24** | **yes** | GLH | Paracentrobia | COI | PCOI_2 | MG904939 | S1A | N 14.22921 E 121.33537 |
|  |  |  |  |  |  |  |  |  |
| **Oligosita_28S_1** | **yes** | BPH | Oligosita | 28S | O28S_1 | MG785491 | S8C | N 14.148391 E 121.322570 |
| **Oligosita_28S_2** | **yes** | BPH | Oligosita | 28S | O28S_2 | MG785492 | S7C | N 14.185338 E 121.360673 |
| **Oligosita_28S_3** | **yes** | BPH | Oligosita | 28S | O28S_3 | MG785493 | S2C | N 14.11540 E 121.41070 |
| **Oligosita_28S_4** | **yes** | BPH | Oligosita | 28S | O28S_4 | MG785494 | S1C | N 14.22857 E121.33542 |
| **Oligosita_28S_5** | **yes** | BPH | Oligosita | 28S | O28S_5 | MG785495 | S8C | N 14.148391 E 121.322570 |
| **Oligosita_28S_6** | **yes** | BPH | Oligosita | 28S | O28S_6 | MG785496 | S6C | N 14.209896 E 121.331937 |
| **Oligosita_28S_7** | **yes** | BPH | Oligosita | 28S | O28S_7 | MG785497 | S5C | N 14.18912 E 121.36439 |
| **Oligosita_28S_8** | **yes** | BPH | Oligosita | 28S | O28S_8 | MG785498 | S5C | N 14.18912 E 121.36439 |
| **Oligosita_28S_9** | **yes** | BPH | Oligosita | 28S | O28S_9 | MG785499 | S3B | N 14.21577 E 121.33724 |
| **Oligosita_28S_10** | **yes** | BPH | Oligosita | 28S | O28S_10 | MG785500 | S3B | N 14.21577 E 121.33724 |
| **Oligosita_28S_11** | **yes** | BPH | Oligosita | 28S | O28S_11 | MG785501 | S8B | N 14.147272 E 121.323637 |
| **Oligosita_28S_14** | **yes** | BPH | Oligosita | 28S | O28S_12 | MG785502 | S1B | N 14.22857 E121.33542 |
| **Oligosita_28S_15** | **yes** | BPH | Oligosita | 28S | O28S_13 | MG785503 | S3B | N 14.21577 E 121.33724 |
| **Oligosita_28S_16** | **yes** | BPH | Oligosita | 28S | O28S_14 | MG785504 | S5B | N 14.18674 E 121.36550 |
| **Oligosita_28S_17** | **yes** | BPH | Oligosita | 28S | O28S_15 | MG785505 | S4A | N 14.13992 E 121.39901 |
| **Oligosita_28S_19** | **yes** | BPH | Oligosita | 28S | O28S_16 | MG785506 | S7A | N 14.184159 E 121.362021 |
| **Oligosita_28S_20** | **yes** | BPH | Oligosita | 28S | O28S_17 | MG785507 | S1A | N 14.22921 E 121.33537 |
| **Oligosita_28S_21** | **yes** | BPH | Oligosita | 28S | O28S_18 | MG785508 | S5A | N 14.18689 E 121.36572 |
| **Oligosita_28S_22** | **yes** | BPH | Oligosita | 28S | O28S_19 | MG785509 | S2A | N 14.11583 E 121.41298 |
| **Oligosita_28S_23** | **yes** | BPH | Oligosita | 28S | O28S_20 | MG785510 | S6A | N 14.207742 E 121.332669 |
|  |  |  |  |  |  |  |  |  |
| **Oligosita_COI_1** | **yes** | BPH | Oligosita | COI | OCOI_1 | MG904893 | S8C | N 14.148391 E 121.322570 |
| **Oligosita_COI_2** | **yes** | BPH | Oligosita | COI | OCOI_1 | MG904894 | S7C | N 14.185338 E 121.360673 |
| **Oligosita_COI_3** | **yes** | BPH | Oligosita | COI | OCOI_1 | MG904895 | S2C | N 14.11540 E 121.41070 |
| **Oligosita_COI_4** | **yes** | BPH | Oligosita | COI | OCOI_1 | MG904896 | S1C | N 14.22857 E121.33542 |
| **Oligosita_COI_5** | **yes** | BPH | Oligosita | COI | OCOI_1 | MG904897 | S8C | N 14.148391 E 121.322570 |
| **Oligosita_COI_6** | **yes** | BPH | Oligosita | COI | OCOI_1 | MG904898 | S6C | N 14.209896 E 121.331937 |
| Oligosita_COI_6b | no | BPH | Oligosita | COI | OCOI_1 | MG904899 | S8C | N 14.148391 E 121.322570 |
| **Oligosita_COI_7** | **yes** | BPH | Oligosita | COI | OCOI_1 | MG904900 | S5C | N 14.18912 E 121.36439 |
| Oligosita_COI_7b | no | BPH | Oligosita | COI | OCOI_1 | MG904901 | S3C | N 14.21740 E 121.33510 |
| **Oligosita_COI_8** | **yes** | BPH | Oligosita | COI | OCOI_1 | MG904902 | S5C | N 14.18912 E 121.36439 |
| **Oligosita_COI_9** | **yes** | BPH | Oligosita | COI | OCOI_1 | MG904903 | S3B | N 14.21577 E 121.33724 |
| **Oligosita_COI_10** | **yes** | BPH | Oligosita | COI | OCOI_1 | MG904904 | S3B | N 14.21577 E 121.33724 |
| **Oligosita_COI_11** | **yes** | BPH | Oligosita | COI | OCOI_1 | MG904905 | S8B | N 14.147272 E 121.323637 |
| Oligosita_COI_11b | no | BPH | Oligosita | COI | OCOI_1 | MG904906 | S8B | N 14.147272 E 121.323637 |
| Oligosita_COI_12 | no | BPH | Oligosita | COI | OCOI_1 | MG904907 | S3B | N 14.21577 E 121.33724 |
| Oligosita_COI_13 | no | BPH | Oligosita | COI | OCOI_1 | MG904908 | S6B | N 14.207981 E 121.332345 |
| **Oligosita_COI_14** | **yes** | BPH | Oligosita | COI | OCOI_1 | MG904909 | S1B | N 14.22857 E121.33542 |
| **Oligosita_COI_15** | **yes** | BPH | Oligosita | COI | OCOI_1 | MG904910 | S3B | N 14.21577 E 121.33724 |
| **Oligosita_COI_16** | **yes** | BPH | Oligosita | COI | OCOI_1 | MG904911 | S5B | N 14.18674 E 121.36550 |
| **Oligosita_COI_17** | **yes** | BPH | Oligosita | COI | OCOI_1 | MG904912 | S4A | N 14.13992 E 121.39901 |
| **Oligosita_COI_19** | **yes** | BPH | Oligosita | COI | OCOI_1 | MG904913 | S7A | N 14.184159 E 121.362021 |
| **Oligosita_COI_20** | **yes** | BPH | Oligosita | COI | OCOI_2 | MG904914 | S1A | N 14.22921 E 121.33537 |
| **Oligosita_COI_21** | **yes** | BPH | Oligosita | COI | OCOI_1 | MG904915 | S5A | N 14.18689 E 121.36572 |
| **Oligosita_COI_22** | **yes** | BPH | Oligosita | COI | OCOI_1 | MG904916 | S2A | N 14.11583 E 121.41298 |
| **Oligosita_COI_23** | **yes** | BPH | Oligosita | COI | OCOI_3 | MG904917 | S6A | N 14.207742 E 121.332669 |
|  |  |  |  |  |  |  |  |  |
| Anagrus_28S_1 | no | BPH | Anagrus | 28S | A28S_1 | MG785407 | S6C | N 14.209896 E 121.331937 |
| **Anagrus_28S_2** | **yes** | BPH | Anagrus | 28S | A28S_2 | MG785408 | S6C | N 14.209896 E 121.331937 |
| **Anagrus_28S_3** | **yes** | BPH | Anagrus | 28S | A28S_2 | MG785409 | S6C | N 14.209896 E 121.331937 |
| Anagrus_28S_4 | **yes** | BPH | Anagrus | 28S | A28S_3 | MG785410 | S4C | N 14.13856 E 121.40070 |
| **Anagrus_28S_5** | **yes** | BPH | Anagrus | 28S | A28S_4 | MG785411 | S6C | N 14.209896 E 121.331937 |
| **Anagrus_28S_6** | **yes** | BPH | Anagrus | 28S | A28S_5 | MG785412 | S2C | N 14.11540 E 121.41070 |
| **Anagrus_28S_7** | **yes** | BPH | Anagrus | 28S | A28S_6 | MG785413 | S6C | N 14.209896 E 121.331937 |
| **Anagrus_28S_9** | **yes** | BPH | Anagrus | 28S | A28S_6 | MG785414 | S5B | N 14.18674 E 121.36550 |
| Anagrus_28S_9b | no | BPH | Anagrus | 28S | A28S_6 | MG785415 | S3B | N 14.21577 E 121.33724 |
| Anagrus_28S_12 | no | BPH | Anagrus | 28S | A28S_5 | MG785416 | S2B | N 14.11495 E 121.41266 |
| **Anagrus_28S_13** | **yes** | BPH | Anagrus | 28S | A28S_6 | MG785417 | S8B | N 14.147272 E 121.323637 |
| **Anagrus_28S_15** | **yes** | BPH | Anagrus | 28S | A28S_5 | MG785418 | S8B | N 14.147272 E 121.323637 |
| **Anagrus_28S_16** | **yes** | BPH | Anagrus | 28S | A28S_5 | MG785419 | S8B | N 14.147272 E 121.323637 |
| **Anagrus_28S_18** | **yes** | BPH | Anagrus | 28S | A28S_7 | MG785420 | S3A | N 14.21248 E 121.33754 |
| **Anagrus_28S_19** | **yes** | BPH | Anagrus | 28S | A28S_5 | MG785421 | S1A | N 14.22921 E 121.33537 |
| **Anagrus_28S_20** | **yes** | BPH | Anagrus | 28S | A28S_6 | MG785422 | S8A | N 14.146850 E 121.323753 |
| Anagrus_28S_21 | **yes** | BPH | Anagrus | 28S | A28S_5 | MG785423 | S5A | N 14.18689 E 121.36572 |
| Anagrus_28S_21b | no | BPH | Anagrus | 28S | A28S_6 | MG785424 | S4A | N 14.13992 E 121.39901 |
| **Anagrus_28S_24** | **yes** | BPH | Anagrus | 28S | A28S_6 | MG785425 | S6A | N 14.207742 E 121.332669 |
|  |  |  |  |  |  |  |  |  |
| Anarus_COI_1 | **yes** | BPH | Anagrus | COI | ACOI_1 | MG911990 | S6C | N 14.209896 E 121.331937 |
| **Anarus_COI_2** | **yes** | BPH | Anagrus | COI | ACOI_2 | MG911991 | S6C | N 14.209896 E 121.331937 |
| **Anarus_COI_3** | **yes** | BPH | Anagrus | COI | ACOI_3 | MG911992 | S6C | N 14.209896 E 121.331937 |
| Anagrus_COI_3b | no | BPH | Anagrus | COI | ACOI_4 | MG911993 | S1C | N 14.22862 E 121.33980 |
| **Anagrus_COI_5** | **yes** | BPH | Anagrus | COI | ACOI_5 | MG911994 | S6C | N 14.209896 E 121.331937 |
| **Anagrus_COI_6** | **yes** | BPH | Anagrus | COI | ACOI_6 | MG911995 | S2C | N 14.11540 E 121.41070 |
| Anagrus_COI_8 | **yes** | BPH | Anagrus | COI | ACOI_7 | MG911996 | S6C | N 14.209896 E 121.331937 |
| **Anagrus_COI_9** | **yes** | BPH | Anagrus | COI | ACOI_8 | MG911997 | S5B | N 14.18674 E 121.36550 |
| Anarus_COI_11 | **yes** | BPH | Anagrus | COI | ACOI_9 | MG911998 | S5B | N 14.18674 E 121.36550 |
| Anagrus_COI_11b | no | BPH | Anagrus | COI | ACOI_9 | MG911999 | S7B | N 14.184277 E 121.361807 |
| **Anagrus_COI_13** | **yes** | BPH | Anagrus | COI | ACOI_9 | MG912000 | S8B | N 14.147272 E 121.323637 |
| Anagrus_COI_14 | **yes** | BPH | Anagrus | COI | ACOI_10 | MG912001 | S2B | N 14.11495 E 121.41266 |
| **Anagrus_COI_15** | **yes** | BPH | Anagrus | COI | ACOI_5 | MG912002 | S8B | N 14.147272 E 121.323637 |
| **Anagrus_COI_16** | **yes** | BPH | Anagrus | COI | ACOI_5 | MG912003 | S8B | N 14.147272 E 121.323637 |
| Anagrus_COI_17 | **yes** | BPH | Anagrus | COI | ACOI_11 | MG912004 | S4A | N 14.13992 E 121.39901 |
| **Anagrus_COI_18** | **yes** | BPH | Anagrus | COI | ACOI_5 | MG912005 | S3A | N 14.21248 E 121.33754 |
| **Anagrus_COI_19** | **yes** | BPH | Anagrus | COI | ACOI_5 | MG912006 | S1A | N 14.22921 E 121.33537 |
| **Anagrus_COI_20** | **yes** | BPH | Anagrus | COI | ACOI_9 | MG912007 | S8A | N 14.146850 E 121.323753 |
| Anagrus_COI_22 | **yes** | BPH | Anagrus | COI | ACOI_9 | MG912008 | S3B | N 14.21577 E 121.33724 |
| Anagrus_COI_23 | **yes** | BPH | Anagrus | COI | ACOI_11 | MG912009 | S3A | N 14.21248 E 121.33754 |
| **Anagrus_COI_24** | **yes** | BPH | Anagrus | COI | ACOI_12 | MG912010 | S6A | N 14.207742 E 121.332669 |
|  |  |  |  |  |  |  |  |  |
| **Gonatocerus_28S_1** | **yes** | GLH | Gonatocerus | 28S | G28S_1 | MG785426 | S2C | N 14.11540 E 121.41070 |
| Gonatocerus_28S_1b | no | GLH | Gonatocerus | 28S | G28S_2 | MG785427 | S2C | N 14.11540 E 121.41070 |
| Gonatocerus_28S_2 | no | GLH | Gonatocerus | 28S | G28S_1 | MG785428 | S8C | N 14.148391 E 121.322570 |
| Gonatocerus_28S_2b | no | GLH | Gonatocerus | 28S | G28S_1 | MG785429 | S8C | N 14.148391 E 121.322570 |
| **Gonatocerus_28S_3** | **yes** | GLH | Gonatocerus | 28S | G28S_3 | MG785430 | S6C | N 14.209896 E 121.331937 |
| Gonatocerus_28S_3b | no | GLH | Gonatocerus | 28S | G28S_1 | MG785431 | S6C | N 14.209896 E 121.331937 |
| Gonatocerus_28S_4 | no | GLH | Gonatocerus | 28S | G28S_1 | MG785432 | S6C | N 14.209896 E 121.331937 |
| Gonatocerus_28S_6 | no | GLH | Gonatocerus | 28S | G28S_1 | MG785433 | S2C | N 14.11540 E 121.41070 |
| **Gonatocerus_28S_7** | **yes** | GLH | Gonatocerus | 28S | G28S_1 | MG785434 | S1C | N 14.22862 E 121.33980 |
| Gonatocerus_28S_7b | no | GLH | Gonatocerus | 28S | G28S_3 | MG785435 | S1C | N 14.22862 E 121.33980 |
| Gonatocerus_28S_8 | no | GLH | Gonatocerus | 28S | G28S_1 | MG785436 | S8C | N 14.148391 E 121.322570 |
| Gonatocerus_28S_8b | no | GLH | Gonatocerus | 28S | G28S_1 | MG785437 | S8C | N 14.148391 E 121.322570 |
| Gonatocerus_28S_9 | no | GLH | Gonatocerus | 28S | G28S_1 | MG785438 | S5B | N 14.18674 E 121.36550 |
| **Gonatocerus_28S_10** | **yes** | GLH | Gonatocerus | 28S | G28S_1 | MG785439 | S5B | N 14.18674 E 121.36550 |
| **Gonatocerus_28S_11** | **yes** | GLH | Gonatocerus | 28S | G28S_1 | MG785440 | S5B | N 14.18674 E 121.36550 |
| **Gonatocerus_28S_12** | **yes** | GLH | Gonatocerus | 28S | G28S_1 | MG785441 | S4B | N 14.13942 E 121.39849 |
| **Gonatocerus_28S_13** | **yes** | GLH | Gonatocerus | 28S | G28S_1 | MG785442 | S6B | N 14.207981 E 121.332345 |
| Gonatocerus_28S_13b | no | GLH | Gonatocerus | 28S | G28S_2 | MG785443 | S6B | N 14.207981 E 121.332345 |
| **Gonatocerus_28S_14** | **yes** | GLH | Gonatocerus | 28S | G28S_1 | MG785444 | S5B | N 14.18674 E 121.36550 |
| Gonatocerus_28S_14b | no | GLH | Gonatocerus | 28S | G28S_2 | MG785445 | S5B | N 14.18674 E 121.36550 |
| **Gonatocerus_28S_15** | **yes** | GLH | Gonatocerus | 28S | G28S_1 | MG785446 | S3B | N 14.21577 E 121.33724 |
| Gonatocerus_28S_15b | no | GLH | Gonatocerus | 28S | G28S_2 | MG785447 | S3B | N 14.21577 E 121.33724 |
| **Gonatocerus_28S_16** | **yes** | GLH | Gonatocerus | 28S | G28S_1 | MG785448 | S4B | N 14.13942 E 121.39849 |
| Gonatocerus_28S_16b | no | GLH | Gonatocerus | 28S | G28S_1 | MG785449 | S4B | N 14.13942 E 121.39849 |
| **Gonatocerus_28S_17** | **yes** | GLH | Gonatocerus | 28S | G28S_4 | MG785450 | S5A | N 14.18689 E 121.36572 |
| **Gonatocerus_28S_18** | **yes** | GLH | Gonatocerus | 28S | G28S_1 | MG785451 | S8A | N 14.146850 E 121.323753 |
| Gonatocerus_28S_18b | no | GLH | Gonatocerus | 28S | G28S_1 | MG785452 | S8A | N 14.146850 E 121.323753 |
| **Gonatocerus_28S_19** | **yes** | GLH | Gonatocerus | 28S | G28S_2 | MG785453 | S3A | N 14.21248 E 121.33754 |
| Gonatocerus_28S_19b | no | GLH | Gonatocerus | 28S | G28S_1 | MG785454 | S4A | N 14.13992 E 121.39901 |
| **Gonatocerus_28S_20** | **yes** | GLH | Gonatocerus | 28S | G28S_1 | MG785455 | S6A | N 14.207742 E 121.332669 |
| **Gonatocerus_28S_21** | **yes** | GLH | Gonatocerus | 28S | G28S_1 | MG785456 | S6A | N 14.207742 E 121.332669 |
| **Gonatocerus_28S_22** | **yes** | GLH | Gonatocerus | 28S | G28S_1 | MG785457 | S3A | N 14.21248 E 121.33754 |
| **Gonatocerus_28S_23** | **yes** | GLH | Gonatocerus | 28S | G28S_3 | MG785458 | S2A | N 14.11583 E 121.41298 |
| **Gonatocerus_28S_24** | **yes** | GLH | Gonatocerus | 28S | G28S_1 | MG785459 | S5A | N 14.18689 E 121.36572 |
|  |  |  |  |  |  |  |  |  |
| **Gonatocerus_COI_1** | **yes** | GLH | Gonatocerus | COI | GCOI_1 | MG904875 | S2C | N 14.11540 E 121.41070 |
| **Gonatocerus_COI_3** | **yes** | GLH | Gonatocerus | COI | GCOI_2 | MG904876 | S6C | N 14.209896 E 121.331937 |
| **Gonatocerus_COI_7** | **yes** | GLH | Gonatocerus | COI | GCOI_1 | MG904877 | S1C | N 14.22862 E 121.33980 |
| **Gonatocerus_COI_10** | **yes** | GLH | Gonatocerus | COI | GCOI_1 | MG904878 | S5B | N 14.18674 E 121.36550 |
| **Gonatocerus_COI_11** | **yes** | GLH | Gonatocerus | COI | GCOI_1 | MG904879 | S5B | N 14.18674 E 121.36550 |
| **Gonatocerus_COI_12** | **yes** | GLH | Gonatocerus | COI | GCOI_1 | MG904880 | S4B | N 14.13942 E 121.39849 |
| **Gonatocerus_COI_13** | **yes** | GLH | Gonatocerus | COI | GCOI_1 | MG904881 | S6B | N 14.207981 E 121.332345 |
| **Gonatocerus_COI_14** | **yes** | GLH | Gonatocerus | COI | GCOI_1 | MG904882 | S5B | N 14.18674 E 121.36550 |
| **Gonatocerus_COI_15** | **yes** | GLH | Gonatocerus | COI | GCOI_1 | MG904883 | S3B | N 14.21577 E 121.33724 |
| **Gonatocerus_COI_16** | **yes** | GLH | Gonatocerus | COI | GCOI_1 | MG904884 | S4B | N 14.13942 E 121.39849 |
| **Gonatocerus_COI_17** | **yes** | GLH | Gonatocerus | COI | GCOI_3 | MG904885 | S5A | N 14.18689 E 121.36572 |
| **Gonatocerus_COI_18** | **yes** | GLH | Gonatocerus | COI | GCOI_1 | MG904886 | S8A | N 14.146850 E 121.323753 |
| **Gonatocerus_COI_19** | **yes** | GLH | Gonatocerus | COI | GCOI_1 | MG904887 | S3A | N 14.21248 E 121.33754 |
| **Gonatocerus_COI_20** | **yes** | GLH | Gonatocerus | COI | GCOI_1 | MG904888 | S6A | N 14.207742 E 121.332669 |
| **Gonatocerus_COI_21** | **yes** | GLH | Gonatocerus | COI | GCOI_1 | MG904889 | S6A | N 14.207742 E 121.332669 |
| **Gonatocerus_COI_22** | **yes** | GLH | Gonatocerus | COI | GCOI_1 | MG904890 | S3A | N 14.21248 E 121.33754 |
| **Gonatocerus_COI_23** | **yes** | GLH | Gonatocerus | COI | GCOI_2 | MG904891 | S2A | N 14.11583 E 121.41298 |
| **Gonatocerus_COI_24** | **yes** | GLH | Gonatocerus | COI | GCOI_1 | MG904892 | S5A | N 14.18689 E 121.36572 |

**Table S3.** Sequences obtained from the NCBI GenBank.

| **Sequence ID** | **Gene** | **Organism** | **Genome (bp)** | **Authors** |
| --- | --- | --- | --- | --- |
| FJ861023.1 | 28S | *Anagrus daanei* | 590 | Triapitsyn,S.V., Rugman-Jones,P.F., Jeong,G., Morse,J.G. and Stouthamer,R. |
| FJ861022.1 | 28S | *Anagrus daanei* | 590 | Triapitsyn,S.V., Rugman-Jones,P.F., Jeong,G., Morse,J.G. and Stouthamer,R. |
| FJ861016.1 | 28S | *Anagrus daanei* | 590 | Triapitsyn,S.V., Rugman-Jones,P.F., Jeong,G., Morse,J.G. and Stouthamer,R. |
| FJ861018.1 | 28S | *Anagrus daanei* | 590 | Triapitsyn,S.V., Rugman-Jones,P.F., Jeong,G., Morse,J.G. and Stouthamer,R. |
| FJ861019.1 | 28S | *Anagrus daanei* | 590 | Triapitsyn,S.V., Rugman-Jones,P.F., Jeong,G., Morse,J.G. and Stouthamer,R. |
| FJ861021.1 | 28S | *Anagrus daanei* | 590 | Triapitsyn,S.V., Rugman-Jones,P.F., Jeong,G., Morse,J.G. and Stouthamer,R. |
| JN623692.1 | 28S | *Anagrus epos* | 1004 | Munro,J.B., Heraty,J.M., Burks,R.A., Hawks,D., Mottern,J., Cruaud,A., Rasplus,J.Y. and Jansta,P. |
| FJ861009.1 | 28S | *Anagrus epos* | 593 | Triapitsyn,S.V., Rugman-Jones,P.F., Jeong,G., Morse,J.G. and Stouthamer,R. |
| FJ861032.1 | 28S | *Anagrus erythroneurae* | 603 | Triapitsyn,S.V., Rugman-Jones,P.F., Jeong,G., Morse,J.G. and Stouthamer,R. |
| FJ861031.1 | 28S | *Anagrus erythroneurae* | 603 | Triapitsyn,S.V., Rugman-Jones,P.F., Jeong,G., Morse,J.G. and Stouthamer,R. |
| FJ861025.1 | 28S | *Anagrus tretiakovae* | 596 | Triapitsyn,S.V., Rugman-Jones,P.F., Jeong,G., Morse,J.G. and Stouthamer,R. |
| FJ861024.1 | 28S | *Anagrus tretiakovae* | 596 | Triapitsyn,S.V., Rugman-Jones,P.F., Jeong,G., Morse,J.G. and Stouthamer,R. |
| AJ547625.1 | 28S | *Encarsia californica* | 658 | Polaszek,A., Manzari,S. and Quicke,D.L.J. |
| KF778730.1 | 28S | *Encarsia citrina* | 615 | Abell,K.J., Driesche,R.G., Normak,B.B. and Gwiazdowski,R.A. |
| KF778711.1 | 28S | *Encarsia citrina* | 614 | Abell,K.J., Driesche,R.G., Normak,B.B. and Gwiazdowski,R.A. |
| KF778703.1 | 28S | *Encarsia citrina* | 556 | Abell,K.J., Driesche,R.G., Normak,B.B. and Gwiazdowski,R.A. |
| KF778697.1 | 28S | *Encarsia citrina* | 605 | Abell,K.J., Driesche,R.G., Normak,B.B. and Gwiazdowski,R.A. |
| KF778694.1 | 28S | *Encarsia citrina* | 621 | Abell,K.J., Driesche,R.G., Normak,B.B. and Gwiazdowski,R.A. |
| AF254236.1 | 28S | *Encarsia citrina* | 600 | Babcock,C.S., Heraty,J.M., De Barro,P.J., Driver,F. and Schmidt,S. |
| KF778734.1 | 28S | *Encarsia citrina* | 611 | Abell,K.J., Driesche,R.G., Normak,B.B. and Gwiazdowski,R.A. |
| AJ547626.1 | 28S | *Encarsia dispersa* | 645 | Polaszek,A., Manzari,S. and Quicke,D.L.J. |
| AJ547627.1 | 28S | *Encarsia dispersa* | 639 | Polaszek,A., Manzari,S. and Quicke,D.L.J. |
| AF223375.1 | 28S | *Encarsia formosa* | 634 | Babcock,C.S. and Heraty,J.M. |
| AY359237.1 | 28S | *Encarsia guadeloupae* | 709 | Antony,B., Palaniswami,M.S. and Sugunan,V.S. |
| AY360217.1 | 28S | *Encarsia haitiensis* | 712 | Antony,B., Palaniswami,M.S. and Sugunan,V.S. |
| AJ547630.1 | 28S | *Encarsia hispida* | 643 | Polaszek,A., Manzari,S. and Quicke,D.L.J. |
| GQ423486.1 | 28S | *Encarsia inaron* | 720 | Harris,L., Perlman,S., Hunter,M., White,J. and Kelly,S. |
| AF223366.1 | 28S | *Encarsia meritoria* | 633 | Babcock,C.S. and Heraty,J.M. |
| KC960150.1 | 28S | *Eurytoma arctica* | 538 | Delvare,G., Gebiola,M., Zeiri,A. and Garonna,A.P |
| KC960093.1 | 28S | *Eurytoma asphodeli* | 540 | Delvare,G., Gebiola,M., Zeiri,A. and Garonna,A.P |
| KC960107.1 | 28S | *Eurytoma laricis* | 538 | Delvare,G., Gebiola,M., Zeiri,A. and Garonna,A.P. |
| AY317171.1 | 28S | *Eurytoma manilensis* | 605 | Chen,Y., Xiao,H., Fu,J. and Huang,D.W. |
| KC960109.1 | 28S | *Eurytoma maura* | 538 | Delvare,G., Gebiola,M., Zeiri,A. and Garonna,A.P |
| KC960120.1 | 28S | *Eurytoma morio* | 538 | Delvare,G., Gebiola,M., Zeiri,A. and Garonna,A.P |
| AY317163.1 | 28S | *Eurytoma orchidearum* | 605 | Chen,Y., Xiao,H., Fu,J. and Huang,D.W. |
| JN623671.1 | 28S | *Eurytoma rhois* | 967 | Munro,J.B., Heraty,J.M., Burks,R.A., Hawks,D., Mottern,J., Cruaud,A., Rasplus,J.Y. and Jansta,P. |
| JN623672.1 | 28S | *Eurytoma squamosa* | 966 | Munro,J.B., Heraty,J.M., Burks,R.A., Hawks,D., Mottern,J., Cruaud,A., Rasplus,J.Y. and Jansta,P. |
| KC960171.1 | 28S | *Eurytoma striolata* | 540 | Delvare,G., Gebiola,M., Zeiri,A. and Garonna,A.P. |
| KC960163.1 | 28S | *Eurytoma striolata* | 540 | Delvare,G., Gebiola,M., Zeiri,A. and Garonna,A.P. |
| KC960148.1 | 28S | *Eurytoma striolata* | 540 | Delvare,G., Gebiola,M., Zeiri,A. and Garonna,A.P. |
| KC960145.1 | 28S | *Eurytoma striolata* | 540 | Delvare,G., Gebiola,M., Zeiri,A. and Garonna,A.P. |
| KC960125.1 | 28S | *Eurytoma striolata* | 540 | Delvare,G., Gebiola,M., Zeiri,A. and Garonna,A.P. |
| KC960122.1 | 28S | *Eurytoma striolata* | 540 | Delvare,G., Gebiola,M., Zeiri,A. and Garonna,A.P. |
| KC999994.1 | 28S | *Eurytoma striolata* | 540 | Asma,Z. |
| KC999992.1 | 28S | *Eurytoma striolata* | 541 | Asma,Z. |
| AY317178.1 | 28S | *Eurytoma verticillata* | 600 | Chen,Y., Xiao,H., Fu,J. and Huang,D.W. |
| AY953531.1 | 28S | *Gonatocerus ashmeadi* | 592 | Triapitsyn,S.V., Vickerman,D.B., Heraty,J.M. and Logarzo,G.A. |
| AY953530.1 | 28S | *Gonatocerus ashmeadi* | 592 | Triapitsyn,S.V., Vickerman,D.B., Heraty,J.M. and Logarzo,G.A. |
| GQ374774.1 | 28S | *Gonatocerus ashmeadi* | 3047 | Heraty,J., Ronquist,F., Carpenter,J.M., Hawks,D., Schulmeister,S., Dowling,A.P., Murray,D., Munro,J., Wheeler,W.C., Schiff,N. , Sharkey,M. |
| AY953528.1 | 28S | *Gonatocerus ashmeadi* | 592 | Triapitsyn,S.V., Vickerman,D.B., Heraty,J.M. and Logarzo,G.A. |
| AY953526.1 | 28S | *Gonatocerus ashmeadi* | 592 | Triapitsyn,S.V., Vickerman,D.B., Heraty,J.M. and Logarzo,G.A. |
| AY953525.1 | 28S | *Gonatocerus ashmeadi* | 592 | Triapitsyn,S.V., Vickerman,D.B., Heraty,J.M. and Logarzo,G.A. |
| JN623700.1 | 28S | *Gonatocerus ashmeadi* | 979 | Munro,J.B., Heraty,J.M., Burks,R.A., Hawks,D., Mottern,J., Cruaud,A., Rasplus,J.Y. and Jansta,P. |
| DQ328663.1 | 28S | *Gonatocerus cf. Morrilli* | 595 | Van Oosten,H.H., Hoddle,M.S., Triapytsin,S.V. and Stouthamer,R. |
| DQ328659.1 | 28S | *Gonatocerus cf. morrilli* | 596 | Van Oosten,H.H., Hoddle,M.S., Triapytsin,S.V. and Stouthamer,R. |
| AY953533.1 | 28S | *Gonatocerus fasciatus* | 603 | Triapitsyn,S.V., Vickerman,D.B., Heraty,J.M. and Logarzo,G.A. |
| AY953534.1 | 28S | *Gonatocerus novifasciatus* | 596 | Triapitsyn,S.V., Vickerman,D.B., Heraty,J.M. and Logarzo,G.A. |
| AY953532.1 | 28S | *Gonatocerus triguttatus* | 597 | Triapitsyn,S.V., Vickerman,D.B., Heraty,J.M. and Logarzo,G.A. |
| AY599253.1 | 28S | *Gonatocerus triguttatus* | 985 | Gillespie,J.J., Munro,J.B., Heraty,J.M., Yoder,M.J., Owen,A.K. and Carmichael,A.E. |
| AY953522.1 | 28S | *Gonatocerus uat* | 595 | Triapitsyn,S.V., Vickerman,D.B., Heraty,J.M. and Logarzo,G.A. |
| AY953523.1 | 28S | *Gonatocerus uat* | 595 | Triapitsyn,S.V., Vickerman,D.B., Heraty,J.M. and Logarzo,G.A. |
| AY623555.1 | 28S | *Ittys ceresarum* | 966 | Gillespie,J.J., Munro,J.B., Heraty,J.M., Yoder,M.J., Owen,A.K. and Carmichael,A.E. |
| GQ374773.1 | 28S | *Mymaromma anomalum* | 3141 | Heraty,J., Ronquist,F., Carpenter,J.M., Hawks,D., Schulmeister,S., Dowling,A.P., Murray,D., Munro,J., Wheeler,W.C., Schiff,N. , Sharkey,M. |
| GQ374773.1 | 28S | *Mymaromella mira* | 2995 | Heraty,J., Ronquist,F., Carpenter,J.M., Hawks,D., Schulmeister,S., Dowling,A.P., Murray,D., Munro,J., Wheeler,W.C., Schiff,N. , Sharkey,M. |
| KT124387.1 | 28S | *Oligosita balcluthae* | 550 | Bella,S., Cupani,S., D'urso,V., Laudonia,S., Sinno,M. and Viggiani,G. |
| AY623572.1 | 28S | *Paracentrobia nr. acuminata* | 958 | Gillespie,J.J., Munro,J.B., Heraty,J.M., Yoder,M.J., Owen,A.K. and Carmichael,A.E. |
| AY599406.1 | 28S | *Trichogramma fuentesi* | 964 | Gillespie,J.J., Munro,J.B., Heraty,J.M., Yoder,M.J., Owen,A.K. and Carmichael,A.E. |
| AY623512.1 | 28S | *Trichogramma funiculatum* | 954 | Gillespie,J.J., Munro,J.B., Heraty,J.M., Yoder,M.J., Owen,A.K. and Carmichael,A.E. |
| AY623514.1 | 28S | *Trichogramma minutum* | 964 | Gillespie,J.J., Munro,J.B., Heraty,J.M., Yoder,M.J., Owen,A.K. and Carmichael,A.E. |
| AY623513.1 | 28S | *Trichogramma parkeri* | 962 | Gillespie,J.J., Munro,J.B., Heraty,J.M., Yoder,M.J., Owen,A.K. and Carmichael,A.E. |
| AY599407.1 | 28S | *Trichogramma platneri* | 963 | Gillespie,J.J., Munro,J.B., Heraty,J.M., Yoder,M.J., Owen,A.K. and Carmichael,A.E. |
| AY599408.1 | 28S | *Trichogramma pretiosum* | 963 | Gillespie,J.J., Munro,J.B., Heraty,J.M., Yoder,M.J., Owen,A.K. and Carmichael,A.E. |
| AY623510.1 | 28S | *Trichogrammatoidea bactrae* | 951 | Gillespie,J.J., Munro,J.B., Heraty,J.M., Yoder,M.J., Owen,A.K. and Carmichael,A.E. |
| AY623541.1 | 28S | *Ufensia minuta voucher* | 965 | Gillespie,J.J., Munro,J.B., Heraty,J.M., Yoder,M.J., Owen,A.K. and Carmichael,A.E. |
| EU015027.1 | COI | *Anagrus atomus* | 518 | de Leon,J.H., Triapitsyn,S.V., Matteucig,G., Viggiani,G. and Gonzalez,M. |
| EU015031.1 | COI | *Anagrus ustulatus* | 518 | de Leon,J.H., Triapitsyn,S.V., Matteucig,G., Viggiani,G. and Gonzalez,M. |
| EU015032.1 | COI | *Anagrus ustulatus* | 518 | de Leon,J.H., Triapitsyn,S.V., Matteucig,G., Viggiani,G. and Gonzalez,M. |
| EU015033.1 | COI | *Anagrus ustulatus* | 518 | de Leon,J.H., Triapitsyn,S.V., Matteucig,G., Viggiani,G. and Gonzalez,M. |
| EU015034.1 | COI | *Anagrus ustulatus* | 518 | de Leon,J.H., Triapitsyn,S.V., Matteucig,G., Viggiani,G. and Gonzalez,M. |
| EU015035.1 | COI | *Anagrus ustulatus* | 518 | de Leon,J.H., Triapitsyn,S.V., Matteucig,G., Viggiani,G. and Gonzalez,M. |
| EU015036.1 | COI | *Anagrus ustulatus* | 518 | de Leon,J.H., Triapitsyn,S.V., Matteucig,G., Viggiani,G. and Gonzalez,M. |
| EU015037.1 | COI | *Anagrus ustulatus* | 518 | de Leon,J.H., Triapitsyn,S.V., Matteucig,G., Viggiani,G. and Gonzalez,M. |
| EU015038.1 | COI | *Anagrus ustulatus* | 518 | de Leon,J.H., Triapitsyn,S.V., Matteucig,G., Viggiani,G. and Gonzalez,M. |
| EU015039.1 | COI | *Anagrus ustulatus* | 518 | de Leon,J.H., Triapitsyn,S.V., Matteucig,G., Viggiani,G. and Gonzalez,M. |
| EU015040.1 | COI | *Anagrus ustulatus* | 518 | de Leon,J.H., Triapitsyn,S.V., Matteucig,G., Viggiani,G. and Gonzalez,M. |
| DQ922739.1 | COI | *Anagrus erythroneurae* | 519 | de Leon,J.H., Triapitsyn,S.V., Matteucig,G., Viggiani,G. and Gonzalez,M. |
| HQ599571.1 | COI | *Aphelinus varipes* | 653 | Gariepy,T.D. and Messing,R.H. |
| JQ268916.1 | COI | *Aphytis melinus* | 688 | Jamalomidi,A. |
| JQ268913.1 | COI | *Aphytis hispanicus* | 678 | Jamalomidi,A. and Hosseini,R |
| KR790709.1 | COI | *Closterocerus trifasciatus voucher* | 652 | Hebert,P.D., Ratnasingham,S., Zakharov,E.V., Telfer,A.C., Levesque-Beaudin,V., Milton,M.A., Pedersen,S., Jannetta,P. and deWaard,J.R. |
| GQ922198.1 | COI | *Encarsia diaspidicola* | 518 | De Leon,J.H., Neumann,G., Follett,P.A. and Hollingsworth,R.G. |
| GQ922196.1 | COI | *Encarsia diaspidicola* | 518 | De Leon,J.H., Neumann,G., Follett,P.A. and Hollingsworth,R.G. |
| KF055395.1 | COI | *Encarsia inaron* | 641 | Fattah-Hosseini,S., Karimi,J., Allahyari,H. and Pahlevan, Hashemi,S.H. |
| KF055396.1 | COI | *Encarsia inaron* | 621 | Fattah-Hosseini,S., Karimi,J., Allahyari,H. and Pahlevan, Hashemi,S.H. |
| KF055394.1 | COI | *Encarsia inaron* | 641 | Fattah-Hosseini,S., Karimi,J., Allahyari,H. and Pahlevan Hashemi,S.H. |
| KF055393.1 | COI | *Encarsia inaron* | 633 | Fattah-Hosseini,S., Karimi,J., Allahyari,H. and Pahlevan Hashemi,S.H. |
| KF055392.1 | COI | *Encarsia inaron* | 630 | Fattah-Hosseini,S., Karimi,J., Allahyari,H. and Pahlevan Hashemi,S.H. |
| KF055391.1 | COI | *Encarsia inaron* | 627 | Fattah-Hosseini,S., Karimi,J., Allahyari,H. and Pahlevan Hashemi,S.H. |
| KF055390.1 | COI | *Encarsia inaron* | 633 | Fattah-Hosseini,S., Karimi,J., Allahyari,H. and Pahlevan Hashemi,S.H. |
| KF055389.1 | COI | *Encarsia inaron* | 624 | Fattah-Hosseini,S., Karimi,J., Allahyari,H. and Pahlevan Hashemi,S.H. |
| JQ268914.1 | COI | *Encarsia inquirenda* | 670 | Jamalomidi,A. and Hosseini,R. |
| JF750717.1 | COI | *Encarsia iris* | 651 | Schmidt,S., De Barro,P. and Jamieson,L. |
| JF750718.1 | COI | *Encarsia iris* | 651 | Schmidt,S., De Barro,P. and Jamieson,L. |
| JQ083715.1 | COI | *Encarsia perniciosi* | 693 | Pina,T., Verdu,M.J., Urbaneja,A. and Sabater-Munoz,B. |
| JQ083717.1 | COI | *Encarsia perniciosi* | 699 | Pina,T., Verdu,M.J., Urbaneja,A. and Sabater-Munoz,B. |
| KC685093.1 | COI | *Eurytoma acuta* | 408 | Zhang,Y.M., Gates,M.W. and Shorthouse,J.D. |
| KC685092.1 | COI | *Eurytoma acuta* | 403 | Zhang,Y.M., Gates,M.W. and Shorthouse,J.D. |
| KC685106.1 | COI | *Eurytoma calcarea* | 408 | Zhang,Y.M., Gates,M.W. and Shorthouse,J.D. |
| KC685105.1 | COI | *Eurytoma calcarea* | 404 | Zhang,Y.M., Gates,M.W. and Shorthouse,J.D. |
| KC685140.1 | COI | *Eurytoma discordans* | 415 | Zhang,Y.M., Gates,M.W. and Shorthouse,J.D. |
| KC685139.1 | COI | *Eurytoma discordans* | 471 | Zhang,Y.M., Gates,M.W. and Shorthouse,J.D. |
| KC685152.1 | COI | *Eurytoma iniquus* | 473 | Zhang,Y.M., Gates,M.W. and Shorthouse,J.D. |
| KC685151.1 | COI | *Eurytoma iniquus* | 465 | Zhang,Y.M., Gates,M.W. and Shorthouse,J.D. |
| KC685189.1 | COI | *Eurytoma longavena* | 415 | Zhang,Y.M., Gates,M.W. and Shorthouse,J.D. |
| KC685188.1 | COI | *Eurytoma longavena* | 490 | Zhang,Y.M., Gates,M.W. and Shorthouse,J.D. |
| KC960043.1 | COI | *Eurytoma maura* | 541 | Delvare,G., Gebiola,M., Zeiri,A. and Garonna,A.P. |
| KC960042.1 | COI | *Eurytoma maura* | 541 | Delvare,G., Gebiola,M., Zeiri,A. and Garonna,A.P. |
| KC960088.1 | COI | *Eurytoma morio* | 541 | Delvare,G., Gebiola,M., Zeiri,A. and Garonna,A.P. |
| KC960087.1 | COI | *Eurytoma morio* | 541 | Delvare,G., Gebiola,M., Zeiri,A. and Garonna,A.P. |
| AY317233.1 | COI | *Eurytoma orchidearum* | 439 | Chen,Y., Xiao,H., Fu,J. and Huang,D.W. |
| EF185156.1 | COI | *Eurytoma rhois* | 433 | Rowley,D.L., Coddington,J.A., Gates,M.W., Norrbom,A.L., Ochoa,R.A., Vandenberg,N.J. and Greenstone,M.H. |
| KC960091.1 | COI | *Eurytoma striolata* | 541 | Delvare,G., Gebiola,M., Zeiri,A. and Garonna,A.P. |
| KC960083.1 | COI | *Eurytoma striolata* | 541 | Delvare,G., Gebiola,M., Zeiri,A. and Garonna,A.P. |
| AY971868.1 | COI | *Gonatocerus annulicornis* | 518 | de Leon,J.H., Jones,W.A., Setamou,M. and Morgan,D.J.W. |
| AY971867.1 | COI | *Gonatocerus annulicornis* | 518 | de Leon,J.H., Jones,W.A., Setamou,M. and Morgan,D.J.W. |
| AY971871.1 | COI | *Gonatocerus ashmeadi* | 518 | de Leon,J.H., Jones,W.A., Setamou,M. and Morgan,D.J.W. |
| AY971870.1 | COI | *Gonatocerus ashmeadi* | 518 | de Leon,J.H., Jones,W.A., Setamou,M. and Morgan,D.J.W. |
| KJ164111.1 | COI | *Gonatocerus morrilli* | 516 | Dewaard,J.R., Dewaard,S.L., Brown,H., Dobbie,I., Ivanova,N., Naik,S., Labbe,R., Levesque-Beaudin,V., Pawlowski,A., Ratnasingham,S.R., Sobel,C., Sones,J., Young,M.R., Zakharov,E.V. and Hebert,P.D.N. |
| KJ086927.1 | COI | *Gonatocerus morrilli* | 588 | Dewaard,J.R., Dewaard,S.L., Brown,H., Dobbie,I., Ivanova,N., Naik,S., Labbe,R., Levesque-Beaudin,V., Pawlowski,A., Ratnasingham,S.R., Sobel,C., Sones,J., Young,M.R., Zakharov,E.V. and Hebert,P.D.N. |
| AY971857.1 | COI | *Gonatocerus morrilli* | 518 | de Leon,J.H., Triapitsyn,S.V., Matteucig,G., Viggiani,G. and Gonzalez,M. |
| AY971856.1 | COI | *Gonatocerus morrilli* | 518 | de Leon,J.H., Triapitsyn,S.V., Matteucig,G., Viggiani,G. and Gonzalez,M. |
| AY971855.1 | COI | *Gonatocerus morrilli* | 518 | de Leon,J.H., Triapitsyn,S.V., Matteucig,G., Viggiani,G. and Gonzalez,M. |
| AY971854.1 | COI | *Gonatocerus morrilli* | 518 | de Leon,J.H., Triapitsyn,S.V., Matteucig,G., Viggiani,G. and Gonzalez,M. |
| AY971853.1 | COI | *Gonatocerus morrilli* | 518 | de Leon,J.H., Triapitsyn,S.V., Matteucig,G., Viggiani,G. and Gonzalez,M. |
| AY971852.1 | COI | *Gonatocerus morrilli* | 518 | de Leon,J.H., Triapitsyn,S.V., Matteucig,G., Viggiani,G. and Gonzalez,M. |
| AY971851.1 | COI | *Gonatocerus morrilli* | 518 | de Leon,J.H., Triapitsyn,S.V., Matteucig,G., Viggiani,G. and Gonzalez,M. |
| AY971850.1 | COI | *Gonatocerus morrilli* | 518 | de Leon,J.H., Triapitsyn,S.V., Matteucig,G., Viggiani,G. and Gonzalez,M. |
| AY971849.1 | COI | *Gonatocerus morrilli* | 518 | de Leon,J.H., Triapitsyn,S.V., Matteucig,G., Viggiani,G. and Gonzalez,M. |
| KR807989.1 | COI | *Gonatocerus morrilli voucher* | 652 | Hebert,P.D., Ratnasingham,S., Zakharov,E.V., Telfer,A.C., Levesque-Beaudin,V., Milton,M.A., Pedersen,S., Jannetta,P. and deWaard,J.R. |
| EU682931.1 | COI | *Gonatocerus tuberculifemur* | 518 | Triapitsyn,S.V., Logarzo,G.A., de Leon,J.H. and Virla,E.G. |
| EU682932.1 | COI | *Gonatocerus tuberculifemur* | 518 | Triapitsyn,S.V., Logarzo,G.A., de Leon,J.H. and Virla,E.G. |
| AY971865.1 | COI | *Gonatocerus walkerjonesi* | 518 | de Leon,J.H., Jones,W.A., Setamou,M. and Morgan,D.J.W. |
| AY971864.1 | COI | *Gonatocerus walkerjonesi* | 518 | de Leon,J.H., Jones,W.A., Setamou,M. and Morgan,D.J.W. |
| HM574048.1 | COI | *Mesopolobus amaenus* | 701 | Kaartinen,R., Stone,G.N., Hearn,J., Lohse,K. and Roslin,T. |
| HM574047.1 | COI | *Mesopolobus amaenus* | 698 | Kaartinen,R., Stone,G.N., Hearn,J., Lohse,K. and Roslin,T. |
| HM574075.1 | COI | *Mesopolobus amaenus* | 698 | Kaartinen,R., Stone,G.N., Hearn,J., Lohse,K. and Roslin,T. |
| HM574070.1 | COI | *Mesopolobus fasciiventris* | 698 | Kaartinen,R., Stone,G.N., Hearn,J., Lohse,K. and Roslin,T. |
| HM574069.1 | COI | *Mesopolobus fasciiventris* | 698 | Kaartinen,R., Stone,G.N., Hearn,J., Lohse,K. and Roslin,T. |
| HM574071.1 | COI | *Mesopolobus fuscipes* | 701 | Kaartinen,R., Stone,G.N., Hearn,J., Lohse,K. and Roslin,T. |
| HM574085.1 | COI | *Mesopolobus lichtensteini* | 698 | Kaartinen,R., Stone,G.N., Hearn,J., Lohse,K. and Roslin,T. |
| HM574084.1 | COI | *Mesopolobus lichtensteini* | 698 | Kaartinen,R., Stone,G.N., Hearn,J., Lohse,K. and Roslin,T. |
| HM574080.1 | COI | *Mesopolobus sericeus* | 699 | Kaartinen,R., Stone,G.N., Hearn,J., Lohse,K. and Roslin,T. |
| HM574076.1 | COI | *Mesopolobus sericeus* | 701 | Kaartinen,R., Stone,G.N., Hearn,J., Lohse,K. and Roslin,T. |
| HM574066.1 | COI | *Mesopolobus tibialis* | 701 | Kaartinen,R., Stone,G.N., Hearn,J., Lohse,K. and Roslin,T. |
| HM574065.1 | COI | *Mesopolobus tibialis* | 701 | Kaartinen,R., Stone,G.N., Hearn,J., Lohse,K. and Roslin,T. |
| HQ107471.1 | COI | *Mesopolobus tortricis* | 657 | Smith,M.A., Eveleigh,E.S., McCann,K.S., Merilo,M.T., McCarthy,P.C. and Van Rooyen,K.I. |
| HQ107470.1 | COI | *Mesopolobus tortricis* | 657 | Smith,M.A., Eveleigh,E.S., McCann,K.S., Merilo,M.T., McCarthy,P.C. and Van Rooyen,K.I. |
| HQ107573.1 | COI | *Mesopolobus verditer* | 651 | Smith,M.A., Eveleigh,E.S., McCann,K.S., Merilo,M.T., McCarthy,P.C. and Van Rooyen,K.I. |
| HQ107574.1 | COI | *Mesopolobus verditer* | 657 | Smith,M.A., Eveleigh,E.S., McCann,K.S., Merilo,M.T., McCarthy,P.C. and Van Rooyen,K.I. |
| GQ374670.1 | COI | *Mymaromma anomalum* | 786 | Heraty,J., Ronquist,F., Carpenter,J.M., Hawks,D., Schulmeister,S., Dowling,A.P., Murray,D., Munro,J., Wheeler,W.C., Schiff,N. , Sharkey,M. |
| DQ177918.1 | COI | *Trichogramma achaeae* | 518 | Fu,H.B. and Cong,B. |
| KP089992.1 | COI | *Trichogramma achaeae* | 439 | Reetha,B., Lalitha,Y., Ballal,C.R., Venkatesan,T., Ankita,G. and Jalali,S.K. |
| KM220523.1 | COI | *Trichogramma brassicae* | 627 | Reetha,B., Lalitha,Y., Ballal,C.R., Venkatesan,T. and Jalali,S.K. |
| KM998974.1 | COI | *Trichogramma brassicae* | 551 | Reetha,B., Lalitha,Y., Ballal,C.R., Venkatesan,T., Ankita,G. and Jalali,S.K. |
| FM210198.1 | COI | *Trichogramma brassicae* | 640 | Pasquer,F., Pfunder,M., Frey,B. and Frey,J.E. |
| DQ177917.1 | COI | *Trichogramma cacoeciae* | 518 | Fu,H.B. and Cong,B. |
| KM242285.1 | COI | *Trichogramma cacoeciae* | 639 | Reetha,B., Lalitha,Y., Ballal,C.R., Venkatesan,T., Jalali,S.K. and Ankita,G. |
| DQ177915.1 | COI | *Trichogramma chilonis* | 518 | Fu,H.B. and Cong,B. |
| KM259632.1 | COI | *Trichogramma chilonis* | 603 | Reetha,B., Lalitha,Y., Ballal,C.R., Venkatesan,T., Jalali,S.K. and Ankita,G. |
| JF776380.1 | COI | *Trichogramma danausicida* | 675 | Nesil,L.B., Prashanth,M. and Jalali,S.K. |
| KM105168.1 | COI | *Trichogramma danausicida* | 632 | Reetha,B., Lalitha,Y., Ballal,C.R., Venkatesan,T. and Jalali,S.K. |
| KC411498.1 | COI | *Trichogramma dendrolimi* | 518 | Dong,H., Xie,L. and Cong,B. |
| KC411497.1 | COI | *Trichogramma dendrolimi* | 518 | Dong,H., Xie,L. and Cong,B. |
| KM105170.1 | COI | *Trichogramma evanescens* | 621 | Reetha,B., Lalitha,Y., Ballal,C.R., Venkatesan,T., Ankita,G. and Jalali,S.K. |
| KP127627.1 | COI | *Trichogramma evanescens* | 617 | Reetha,B., Lalitha,Y., Ballal,C.R., Venkatesan,T., Ankita,G. and Jalali,S.K. |
| KP994544.1 | COI | *Trichogramma japonicum* | 652 | Rukhsana,K. and Sebastian,C.D. |
| KM220522.1 | COI | *Trichogramma japonicum* | 612 | Reetha,B., Lalitha,Y., Ballal,C.R., Venkatesan,T. and Jalali,S.K. |
| KJ083959.1 | COI | *Trichogramma platneri* | 608 | Dewaard,J.R., Dewaard,S.L., Brown,H., Dobbie,I., Ivanova,N., Naik,S., Labbe,R., Levesque-beaudin,V., Pawlowski,A., Ratnasingham,S.R., Sobel,C., Sones,J., Young,M.R., Zakharov,E.V. and Hebert,P.D.N. |
| KJ084938.1 | COI | *Trichogramma platneri* | 612 | Dewaard,J.R., Dewaard,S.L., Brown,H., Dobbie,I., Ivanova,N., Naik,S., Labbe,R., Levesque-beaudin,V., Pawlowski,A., Ratnasingham,S.R., Sobel,C., Sones,J., Young,M.R., Zakharov,E.V. and Hebert,P.D.N. |
| KM998973.1 | COI | *Trichogramma pretiosum* | 553 | Reetha,B., Lalitha,Y., Ballal,C.R., Venkatesan,T., Ankita,G. and Jalali,S.K. |
| KM232609.1 | COI | *Trichogramma pretiosum* | 645 | Reetha,B., Lalitha,Y., Ballal,C.R., Venkatesan,T. and Jalali,S.K. |
| KF573395.1 | COI | *Trichogramma pretiosum* | 652 | Guzman-Larralde,A., Cerna-Chavez,E., Rodriguez-Campos,E., Rugman-Jones,P.F. and Stouthamer,R |
| KF573394.1 | COI | *Trichogramma pretiosum* | 652 | Guzman-Larralde,A., Cerna-Chavez,E., Rodriguez-Campos,E., Rugman-Jones,P.F. and Stouthamer,R |
| KF573406.1 | COI | *Trichogramma pretiosum* | 652 | Guzman-Larralde,A., Cerna-Chavez,E., Rodriguez-Campos,E., Rugman-Jones,P.F. and Stouthamer,R |
| KF573405.1 | COI | *Trichogramma pretiosum* | 652 | Guzman-Larralde,A., Cerna-Chavez,E., Rodriguez-Campos,E., Rugman-Jones,P.F. and Stouthamer,R |
| KF573402.1 | COI | *Trichogramma pretiosum* | 652 | Guzman-Larralde,A., Cerna-Chavez,E., Rodriguez-Campos,E., Rugman-Jones,P.F. and Stouthamer,R |
| KF573401.1 | COI | *Trichogramma pretiosum* | 652 | Guzman-Larralde,A., Cerna-Chavez,E., Rodriguez-Campos,E., Rugman-Jones,P.F. and Stouthamer,R |
| KF573400.1 | COI | *Trichogramma pretiosum* | 652 | Guzman-Larralde,A., Cerna-Chavez,E., Rodriguez-Campos,E., Rugman-Jones,P.F. and Stouthamer,R |
| KF573399.1 | COI | *Trichogramma pretiosum* | 652 | Guzman-Larralde,A., Cerna-Chavez,E., Rodriguez-Campos,E., Rugman-Jones,P.F. and Stouthamer,R |
| KF573398.1 | COI | *Trichogramma pretiosum* | 652 | Guzman-Larralde,A., Cerna-Chavez,E., Rodriguez-Campos,E., Rugman-Jones,P.F. and Stouthamer,R |
| KF573397.1 | COI | *Trichogramma pretiosum* | 652 | Guzman-Larralde,A., Cerna-Chavez,E., Rodriguez-Campos,E., Rugman-Jones,P.F. and Stouthamer,R |
| KP233826.1 | COI | *Trichogrammatoidea robusta* | 617 | Reetha,B., Lalitha,Y., Ballal,C.R., Venkatesan,T. and Jalali,S.K. |


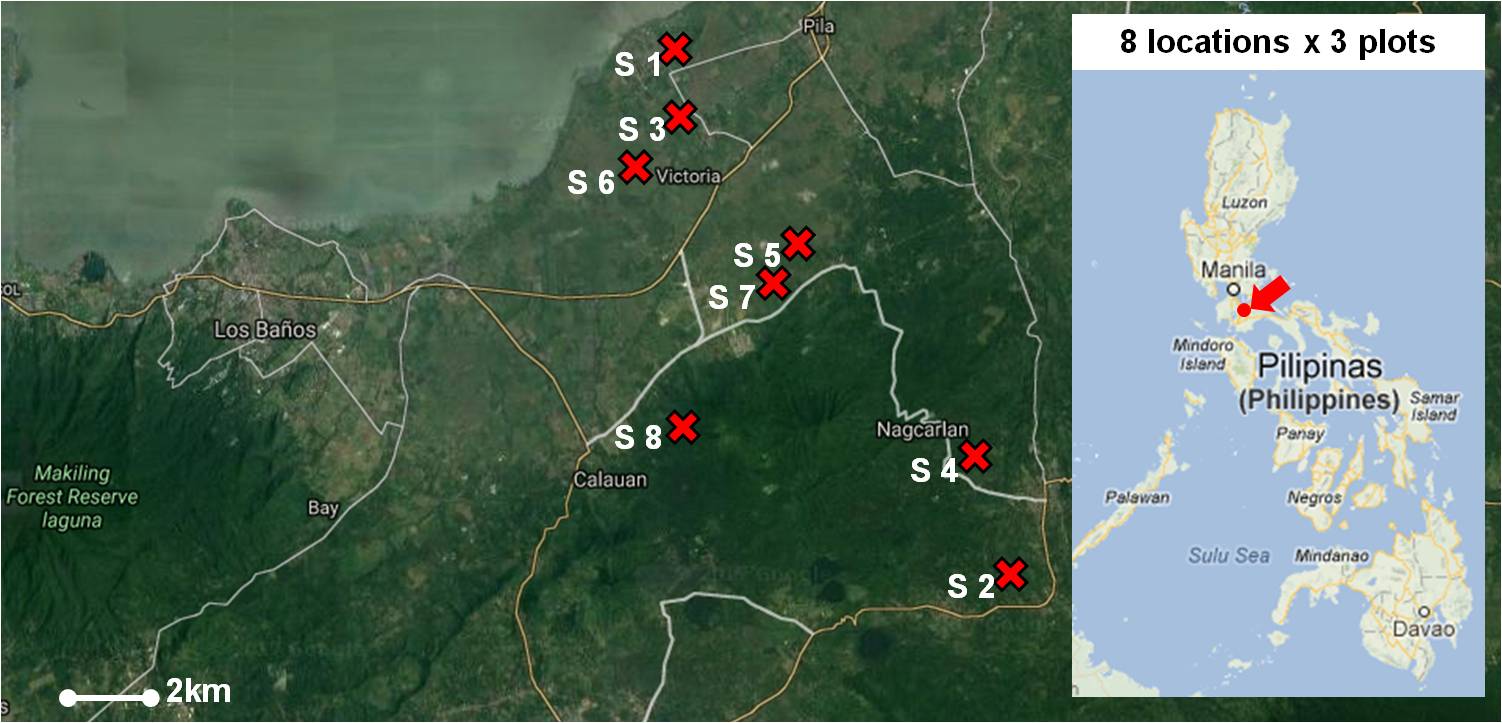
**Figure S1.** Distribution of the eight study sites throughout the Laguna province.


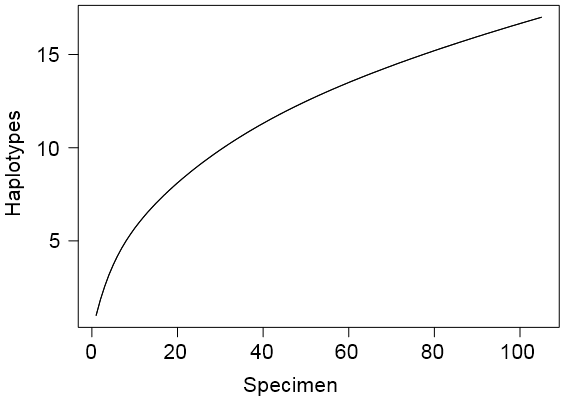


**Figure S2.** Rarefaction curve for the 28S gene fragment.


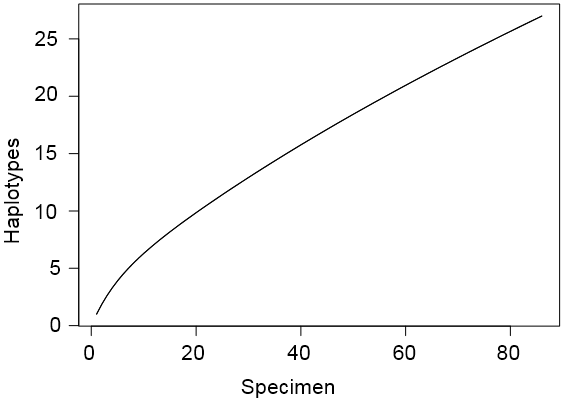


**Figure S3.** Rarefaction curve for the COI gene fragment.


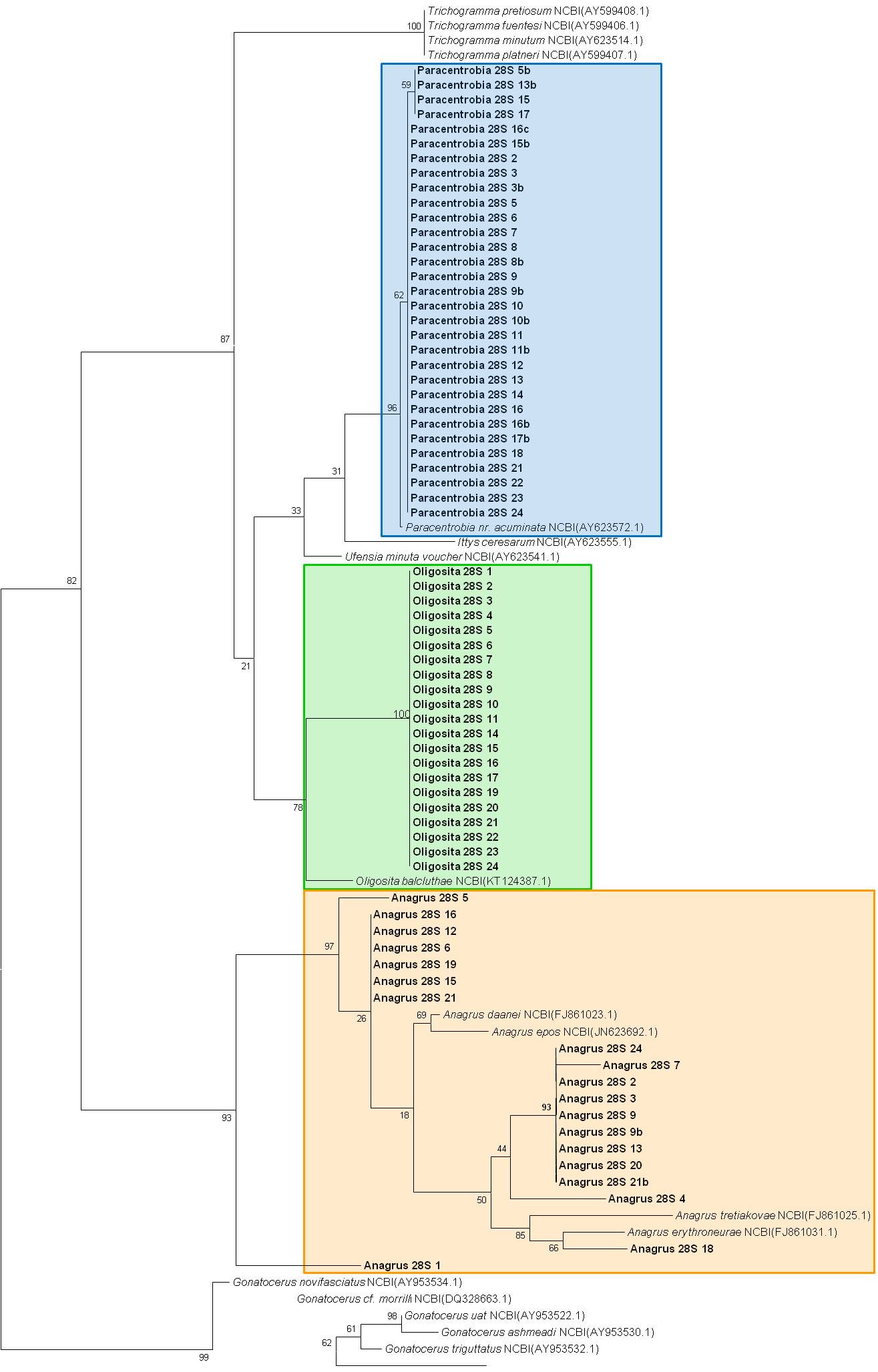


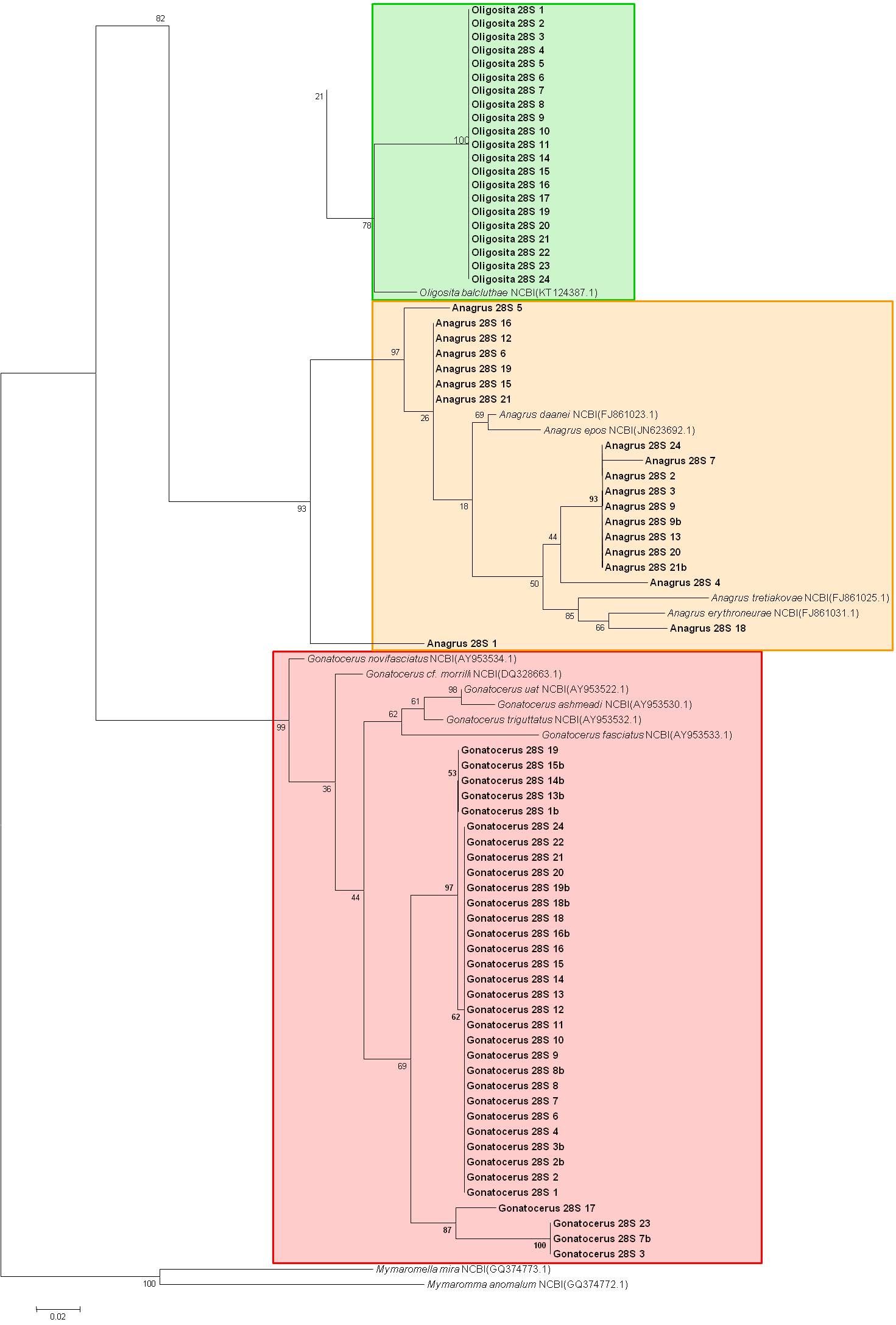


**Figure S4.** Maximum likelihood tree for the 28S sequences, including 105 rice parasitoids and 17 outgroup specimens. Maximum likelihood bootstrap values are given for each node.


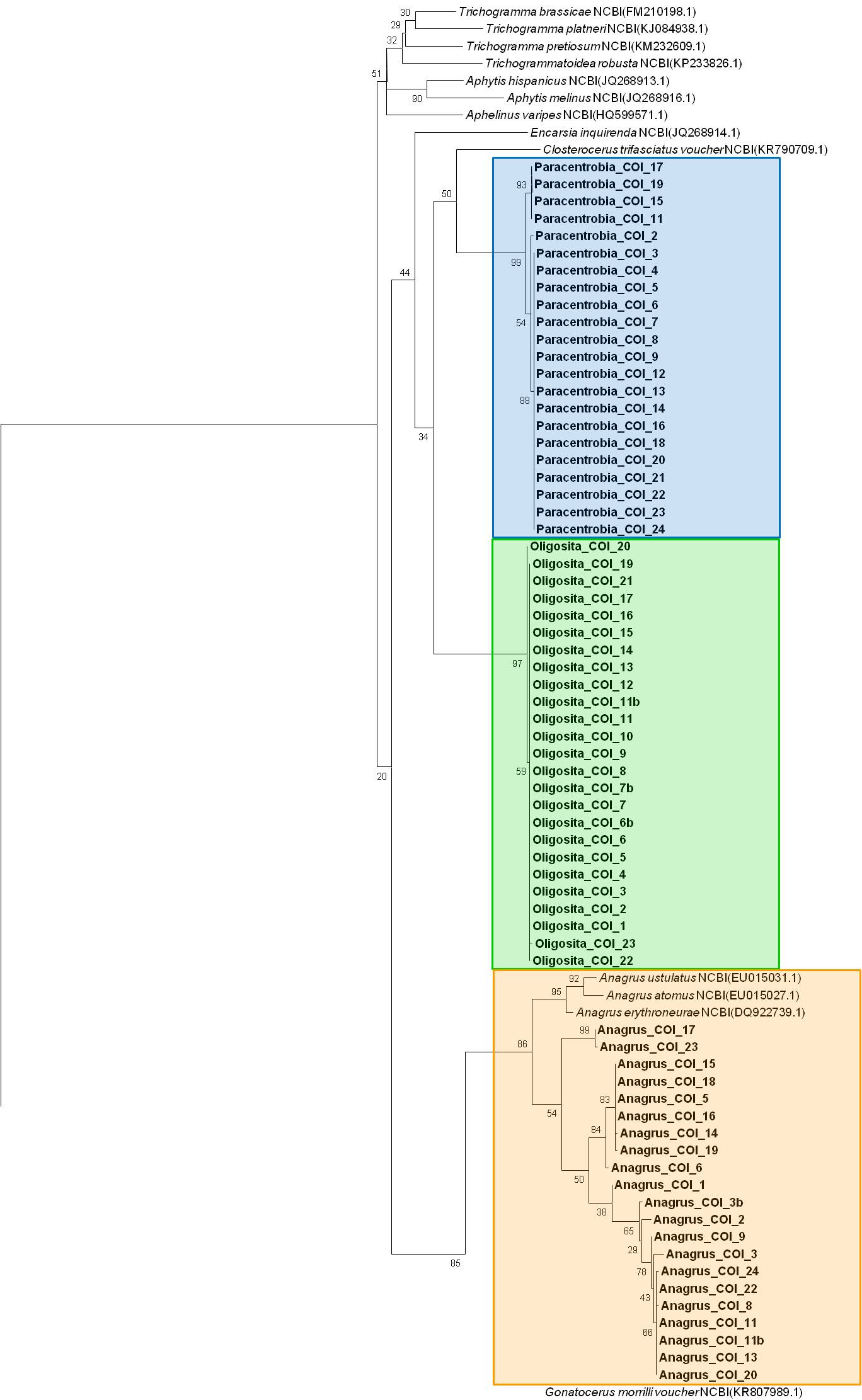


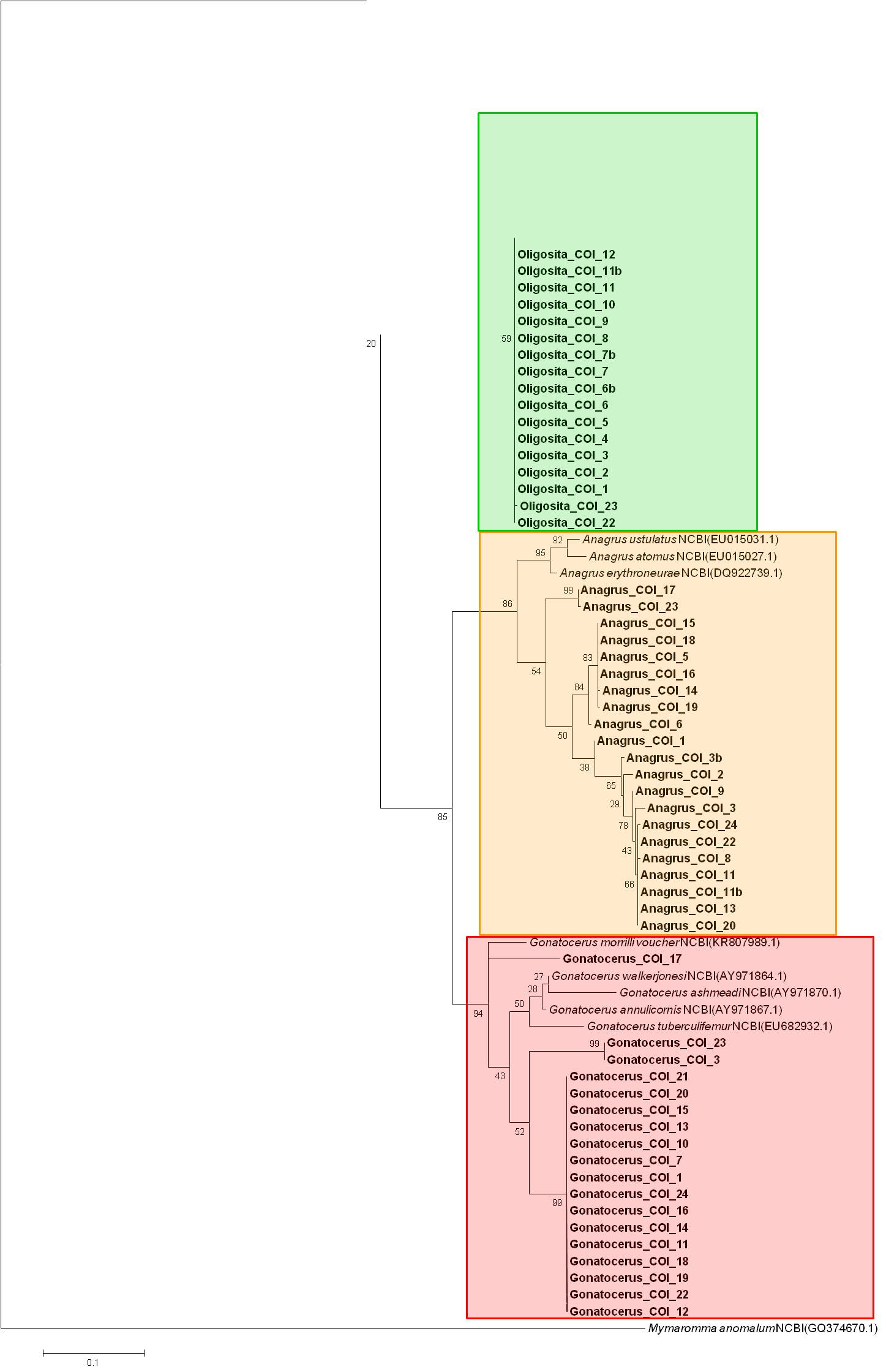


**Figure S5.** Maximum likelihood tree for the COI Sequences, including 85 rice parasitoids and 15 outgroup specimens. Maximum likelihood bootstrap values are given for each node.

© 2018 by the authors. Submitted for possible open access publication under the
terms and conditions of the Creative Commons Attribution (CC BY) license (http://creativecommons.org/licenses/by/4.0/).
